# APOBEC3G-Augmented Stem Cell Therapy to Modulate HIV Replication: A Computational Study

**DOI:** 10.1371/journal.pone.0063984

**Published:** 2013-05-22

**Authors:** Iraj Hosseini, Feilim Mac Gabhann

**Affiliations:** Institute for Computational Medicine, Department of Biomedical Engineering, Johns Hopkins University, Baltimore, Maryland, United States of America; St. Jude Children’s Research Hospital, United States of America

## Abstract

The interplay between the innate immune system restriction factor APOBEC3G and the HIV protein Vif is a key host-retrovirus interaction. APOBEC3G can counteract HIV infection in at least two ways: by inducing lethal mutations on the viral cDNA; and by blocking steps in reverse transcription and viral integration into the host genome. HIV-Vif blocks these antiviral functions of APOBEC3G by impeding its encapsulation. Nonetheless, it has been shown that overexpression of APOBEC3G, or interfering with APOBEC3G-Vif binding, can efficiently block *in vitro* HIV replication. Some clinical studies have also suggested that high levels of APOBEC3G expression in HIV patients are correlated with increased CD4+ T cell count and low levels of viral load; however, other studies have reported contradictory results and challenged this observation. Stem cell therapy to replace a patient’s immune cells with cells that are more HIV-resistant is a promising approach. Pre-implantation gene transfection of these stem cells can augment the HIV-resistance of progeny CD4+ T cells. As a protein, APOBEC3G has the advantage that it can be genetically encoded, while small molecules cannot. We have developed a mathematical model to quantitatively study the effects on *in vivo* HIV replication of therapeutic delivery of CD34+ stem cells transfected to overexpress APOBEC3G. Our model suggests that stem cell therapy resulting in a high fraction of APOBEC3G-overexpressing CD4+ T cells can effectively inhibit *in vivo* HIV replication. We extended our model to simulate the combination of APOBEC3G therapy with other biological activities, to estimate the likelihood of improved outcomes.

## Introduction

The innate immune system is a key line of defense against human immunodeficiency virus type 1 (HIV-1), reducing viral replication and protecting neighboring cells from infection. Key in this battle between host and virus are cytosolic host cell proteins with antiretroviral activities, termed restriction factors. The apolipoprotein B (apo B) messenger RNA (mRNA)-editing, catalytic polypeptide-like 3 (APOBEC3) family of proteins are known to be potent restriction factors and to counteract infection by HIV-1 (reviewed in [1]–[9]). While the seven APOBEC3 proteins have varying levels of potency, in *in vitro* tissue culture APOBEC3G (A3G) exhibits the highest activity against HIV-1 that lacks the viral infectivity factor (*vif*) gene [1], [2].

Ten years after its discovery [10], the antiviral functions of A3G are still the subject of active research. Hypermutation of HIV cDNA via the deaminase functionality of A3G is thought to be the most important A3G mechanism against HIV-1. A3G can induce up to 10% guanosine to adenosine (G-to-A) mutations into viral reverse transcripts [2], [6], [11], by deaminating cytidine (C) to uridine (U) on the minus strand [10], [12]–[15]. This high mutational frequency can destroy viral genome integrity, resulting in production of noninfectious virions. Several groups have suggested that deaminase-independent antiviral activities of A3G also play a role in blocking HIV-1 replication. These include inhibiting several steps during viral reverse transcription and integration [16]–[26]. Note that it is the A3G from the cell in which the virus is made that binds to viral mRNA and gets encapsulated in progeny virions. It is only after the virus is released and infects another cell that the encapsulated A3G exerts both its deaminase-dependent and -independent activities.

As mentioned earlier, HIV-1 has developed the ability to evade the antiviral activities of A3G through the expression of Vif, a viral-encoded protein [27]–[29]. Vif binds to A3G [30]–[33] and exerts multiple counter-mechanisms to block encapsulation of A3G into virions. One mechanism is Vif-induced degradation of A3G where Vif recruits an E3 ubiquitin ligase complex and facilitates degradation of A3G through the proteasomal pathway [10], [34]–[40]. It has also been suggested that Vif directly impedes encapsulation of A3G into virions [30], [37], [41].

**Figure 1:**
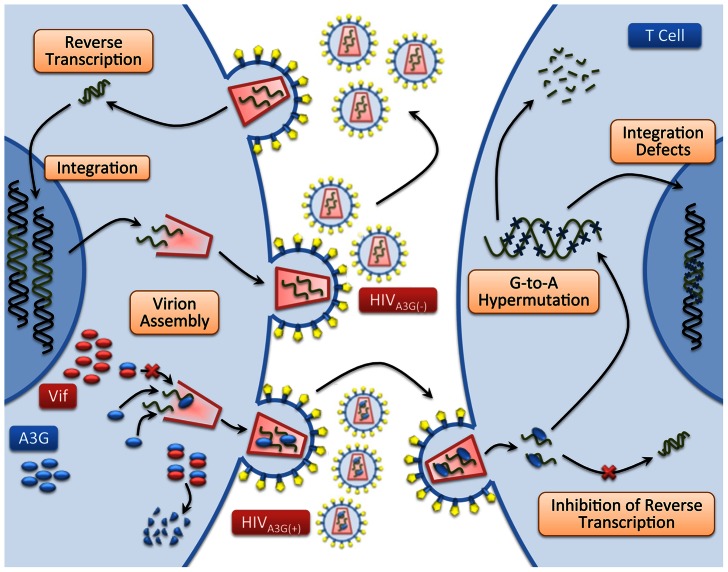
HIV life cycle. Mechanism of HIV infection including viral entry, reverse transcription, integration of viral DNA, virion assembly and release of viral particles is schematically shown. A3G, a host protein and a restriction factor, binds to viral mRNA and gets encapsulated into the viral capsid. If viruses carrying A3G infect other cells, the packaged A3G will exert several antiviral activities, which include inducing G-to-A mutations into viral reverse transcripts by deaminating C to U on the minus strand, blocking multiple steps in reverse transcription and causing integration defects. Vif, a viral protein, binds to A3G and inhibits encapsulation of A3G into virions by facilitating degradation of this protein through the proteasomal pathway.

Fig. 1 shows a basic diagram of HIV infection as well as interactions between HIV-1 and A3G in HIV producing and newly infected CD4+ T cells. We previously developed a multi-scale computational model of HIV infection in *in vitro* T cell culture, consisting of intracellular, cellular and extracellular events [42]. One of the predictions of that model was that overexpression of A3G or of a mutated form lacking the Vif-binding site (termed A3G^ΔVif^) [43], [44] can effectively stop *in vitro* HIV replication. This prediction was in agreement with a number of studies in which elevated levels of A3G expression resulted in A3G overcoming the effects of Vif [10], [41], [45], [46]. The model also predicted that the degradation of A3G by Vif is not a crucial step in HIV pathogenesis; instead it is the binding of A3G to Vif that is the key step and must be targeted to improve A3G efficacy [42]. Our goal in this study is to transpose our validated model of A3G-Vif interactions from simulations of *in vitro* cell culture to simulations of *in vivo* HIV infection and treatment.

Targeting the A3G-Vif pathway may provide a new class of antiretroviral therapy; however, some clinical studies have provided controversial results [47]–[58], and to date, these studies on the effects of A3G on HIV disease progression have not covered large numbers of individuals. In a 2005 study, Jin *et al.* found that in a group of 25 untreated HIV+ patients, A3G mRNA levels were negatively correlated with HIV viral loads and were significantly associated with CD4+ T cell counts [47]. Results reported by two other research groups found that subjects with high G-to-A hypermutation had lower plasma HIV RNA levels and higher CD4+ T cell counts; however A3G mRNA levels were not directly measured [48], [49]. Interestingly, the association of reduced plasma HIV RNA levels with hypermutation was considerably greater than association of reduced plasma HIV RNA levels with the CCR5Δ32 allele [48]. Ulenga *et al.* found that the expression levels of A3G were correlated with the levels of hypermutation in the *vif* and *env* regions, but not in the *gag* region of the virus genome. On the other hand, their study suggested no correlation between plasma viral loads and the levels of hypermutation in the *vif*, *env*, and *gag* regions [50]. In contrast, another study published by the same group found that the expression levels of A3G mRNA in patients with low viral set point were significantly higher than those of patients with high viral set point [51]. While other clinical studies have also shown the ability of A3G to modulate *in vivo* HIV infection [52], [53], some groups have not been able to reproduce the same results [54]–[58]. Therefore, further investigation on the role of A3G in HIV disease progression would greatly benefit the field.

In addition to the A3G-Vif axis being a potential therapeutic anti-HIV approach, recent studies have suggested that A3G may also be used as a preventive strategy against HIV-1. Biasin *et al.* demonstrated that HIV-exposed seronegative subjects had significantly increased A3G mRNA and protein expressions compared to HIV-seropositive patients and healthy control individuals [59]. This higher expression was associated with lower susceptibility of cells to *in vitro* HIV infection [59]. Similar results were reported in a 2009 study by Vázquez-Pérez *et al.*, where the average A3G mRNA expression was over 2-fold higher in exposed uninfected subjects that those of healthy control individuals [52].

Replacing HIV-susceptible cells with more resistant cells, for example, by using gene therapy with or without hematopoietic stem cell transplantation, is proposed as one possible way to achieve a functional cure for HIV infection [60], [61]. A functional cure, as opposed to eradicative cure, is a stable suppression of HIV-1 replication in the absence of highly active antiretroviral therapy [62]. Current advances in the use of gene therapy methods for HIV-1 treatment [63]–[68] and one report of successful eradication of virus in an infected individual [69]–[71] suggest that transfection of CD34+ stem cells with a gene encoding wild type (WT) A3G or its variants will be feasible in the future [1], [72]. Considering that clinical effects of A3G are not clear, we aim to predict the effects of A3G-augmented stem cell therapy (A3G-SCT) on modulation of *in vivo* HIV replication and to quantify some critical hurdles using mathematical models and simulations.

Our central question is, can the transplantation of stem cells transfected with A3G or its variants halt *in vivo* HIV replication? If yes, what percentage of the cells must be transfected and how much inhibitory potency (A3G overexpression), is required?

In this paper, we build upon the basic model of *in vivo* HIV infection [73]–[78] and extend it using the results of our multi-scale model of A3G-Vif interactions (Fig. 1 and [42]). Other relevant biological phenomena such as accelerated cell death are also included into extended models in order to study their impacts on the performance of A3G-SCT. Then we analyze the models at steady state to evaluate the likely long-term effects of this treatment.

## Models

In this section, we briefly introduce the basic model of *in vivo* HIV infection [73]–[78] and then extend it into models capable of studying the effects of A3G-augmented stem cell therapy (A3G-SCT) on virus replication in the body. All variables are capitalized and represent concentrations of different cell types or viruses with units of µl^−1^. The following equations describe the *in vivo* viral dynamics of HIV.
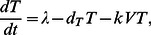
(1a)

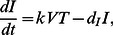
(1b)

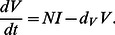
(1c)


**Figure 2:**
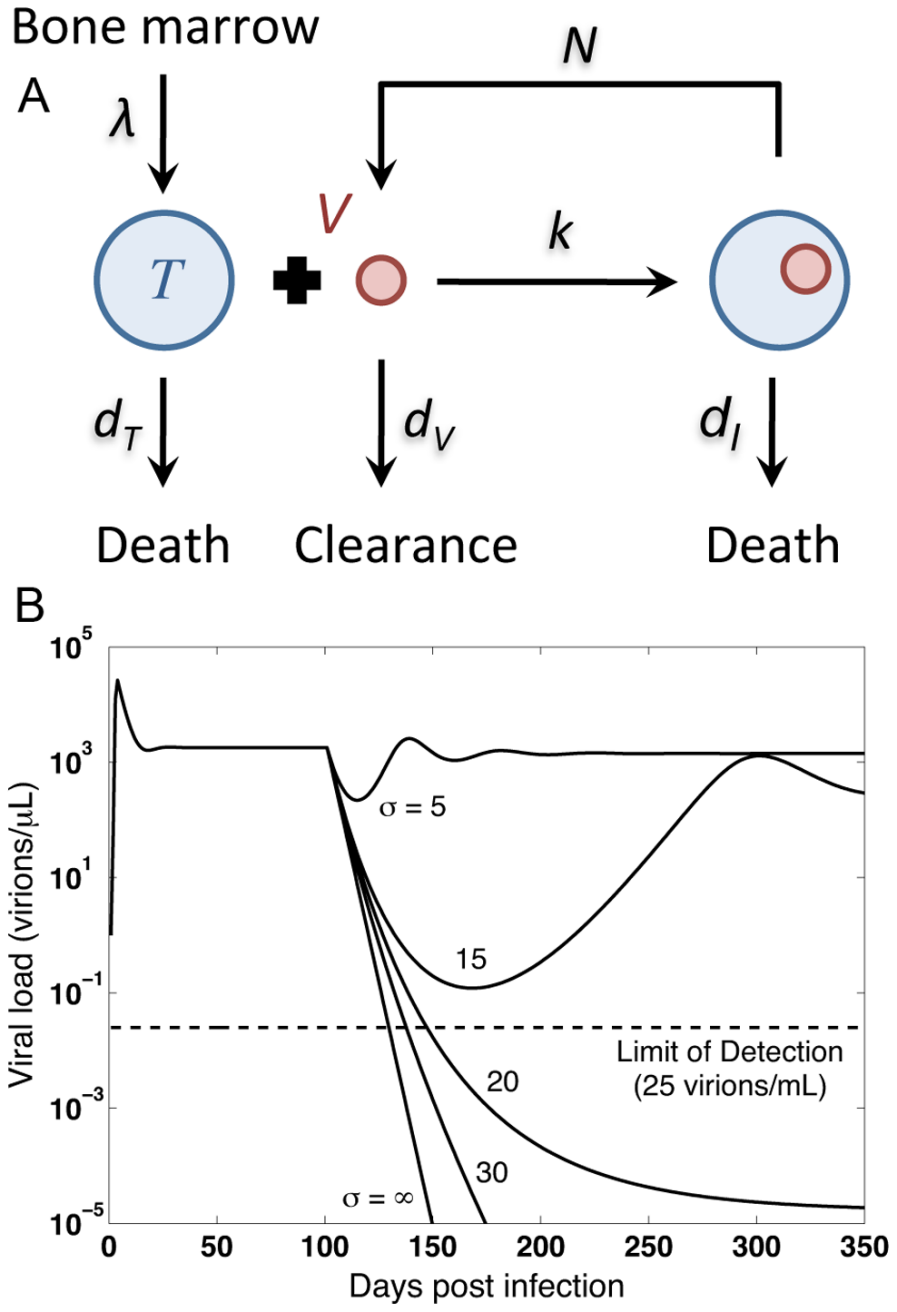
The basic HIV model: schematic diagram and simulations. (A) The model consists of three entities: Free viruses, “uninfected” and “infected” CD4+ T cells. Before infection, only uninfected cells are present with the production rate of *λ* and the death rate of 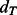. In the model, infection occurs by introducing an initial amount of viruses to the body. Free viruses infect uninfected cells and give rise to infected cells with *k* representing infectivity rate constant. Infected cells die at a rate of 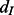; before death, these cells produce and release *N* new free virions per day. The *in vivo* clearance rate of viruses is denoted by 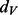. (B) The basic reproductive ratio, *R*_0_, is defined as the number of new infections that arise from a single infected cell when almost all the other cells are uninfected. This important metric determines whether the infection spreads (*R*_0_>1) or dies out (*R*_0_<1) in the body. In the numerical simulation, *R*_0_ = 20 initially and a hypothetical treatment is administered on day 100 which reduces the value of reproductive ratio by *σ* = 5, 15, 20, 30, and ∞. Although *σ* = ∞ results in the fastest decline in the viral load, the gap between curves associated with *σ* = 30 and ∞ is small.

In this model, free viruses infect susceptible CD4+ T cells and give rise to infected cells (Fig. 2A). *T*, *I* and *V* represent the concentration of uninfected CD4+ T cells, infected cells, and free virus, respectively. The rate of infection is proportional to the concentration of free virus and T cells and is equal to *kVT* where *k* is the infectivity rate constant with units µl×day^−1^. The infected cells produce and release on average *N* new free virions per day. Uninfected cells are produced in the thymus at a constant rate, *λ* cells/(µl×day). Uninfected and infected cells die at the rates 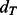 and 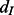, respectively. Free viruses are cleared at a rate of 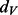. The last three parameters have units of day^−1^. The values for these parameters in our models are consistent with previously published results [42], [73], [77], [79]–[86] and are given in Table 1.

**Table 1:** Parameter values used for simulations and calculations.

Parameter (units)	Values	References	Definition
*λ* (cells×µl^−1^/day)	20	[73], [85]	Production rate of uninfected CD4+ T cells in thymus
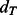 (day^−1^)	0.02	[79], [85]	Death rate of uninfected cells
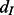 (day^−1^)	0.39	[86]	Death rate of infected cells
*k* (µl×day^−1^)	2.11×10^−4^	Calculated	Rate constant for uninfected cells getting infected by free virus
*N* (virions×day^−1^)	850	[86]	Average number of free virus produced by an infected cell
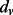 (day^−1^)	23	[77], [81]	Clearance rate of free virus
*p^(wt)^*	0.83	Estimated	A3G-free virus release ratio: fraction of A3G(−) viruses released from infected WT CD4+ T cells
*p* ^(+)^	10^−4^ to10^−1^	[42]	A3G-free virus release ratio: fraction of A3G(−) viruses released from infected A3G-augmented cells
*c*	10^−3^ to10^−2^	[42]	Reduction in the average number of released virions from cells infected by A3G(+) viruses
*f*	0 to 1		Fraction of uninfected CD4+ T cells that overexpress A3G
*t*	>1		Ratio of death rate of infected A3G-augmented cells to death rate of infected WT cells
*w*	>1		Ratio of death rate of cells infected by A3G(+) viruses to death rate of cells infected by A3G(−) viruses
*r*	0 to 1		Apoptosis failure rate: fraction of infected A3G-augmented cells that escape apoptosis
*R*_0_	20	[86]–[88]	Basic reproductive ratio (output): number of new infections arising from a single infected cell when almost all the other cells are uninfected.
*σ*	Varies	Calculated	Reduction factor (output): reduction in the basic reproductive ratio resulting from A3G-SCT

Prior to infection, there are zero viruses and zero infected cells; Uninfected cells are at the steady state value balancing production and death, i.e., . After infection occurs (in the model, this is represented by an initial input of viruses), the system moves towards one of two possible equilibrium points,
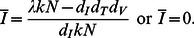
(2)


Where 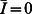 suggests that virus has been eradicated from the body while 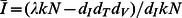 implies that virus has grown and established an infection in the body. The latter equilibrium point is only stable if the concentration of infected cells is greater than zero, meaning that the numerator in equation (2) takes a positive value or
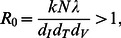
(3)where *R*_0_ is known as the basic reproductive ratio, describing the average number of secondary infected cells arising from each primary infected cell when almost all cells are uninfected. Therefore, if the reproductive ratio takes values greater than one, HIV infection spreads through the body; while values less than one imply that the infection dies out and the virus becomes eradicated. In a study by Little *et al.*, the basic reproductive ratio of 4 patients was estimated in the range of 5.2 to 9.1 with a mean value of 7.1 [87]. Stafford *et al*. analyzed 10 patients and found a median of 5.7, with a range between 2.8 and 11.0 for *R*_0_ [86]. Using a slightly different model, one group found *R*_0_ estimates ranging from 7.4 to 34, with a mean value of 19.3 [87] and recently another group obtained an estimated mean of 8.6 for *R*_0_ with 75% of 47 patients having *R*_0_ values less than 11 and two infected individuals having *R*_0_ values greater than 20 (highest value = 26.4) [88]. The goal of any strategy for HIV treatment or cure must be to reduce *R*_0_ to values less than one. Fig. 2B shows the impact of decreasing *k* and hence reducing *R*_0_ on the system.

### Model I: The Basic HIV Model for A3G-Augmented Cells

In the first extension of the basic model of HIV infection, we assume all the stem cells are transfected with A3G and hence all the progeny CD4+ T cells in the body overexpress A3G in addition to their biological expression of A3G; we term these A3G-augmented cells as opposed to WT cells. When these cells are infected by viruses, A3G overexpressed in cells overcomes Vif and gets encapsulated in some of the budded viruses, meaning that infected cells produce two types of virus: those that carry A3G, hence called A3G(+) viruses, and those viruses that do not, dubbed A3G(−) viruses; the ratio of A3G(−) to total released viruses is an important factor in the model. Cells infected by A3G(+) viruses produce fewer virions compared to cells infected by A3G(−) viruses, because the A3G carried in A3G(+) viruses affects viral production by inhibiting several steps of the HIV life cycle inside the infected cell. The model of HIV infection for A3G-augmented cells can be described by the following equations.
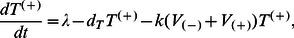
(4a)

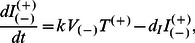
(4b)

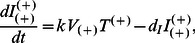
(4c)

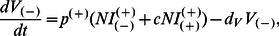
(4d)

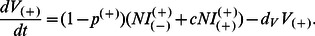
(4e)


Where 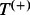 represents the concentration of A3G-augmented CD4+ T cells and 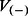 and 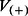 denote the concentration of A3G(−) and A3G(+) viruses, respectively. 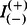 and 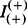 represent the concentration of A3G-augmented cells infected by A3G(−) and A3G(+) viruses, respectively.

**Figure 3:**
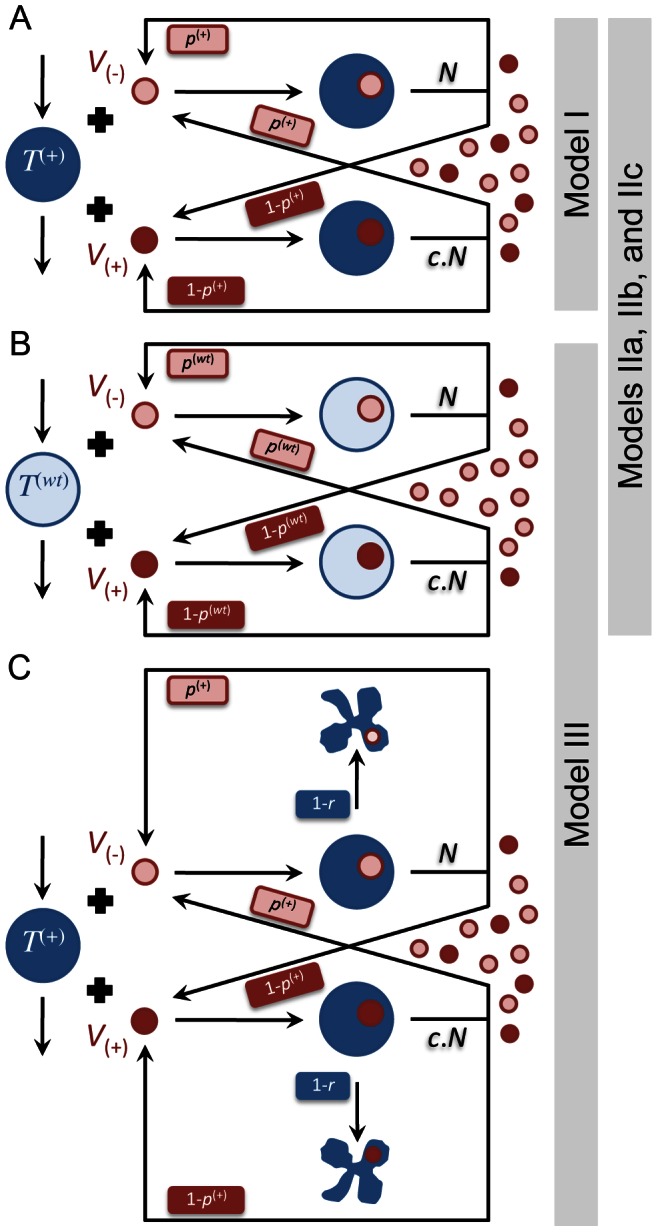
Schematic diagram of the extended models of HIV infection for WT and A3G-augmented cells. Submodels (A) and (B) show schematic diagrams of HIV infection in A3G-augmented and WT cells, represented by dark and light blue large circles, respectively. In all the submodels, HIV infection occurs by a mixed population of A3G(+) and A3G(−) viruses, represented by dark and light red small circles, respectively. Large circles with a small circle inside them represent infected cells with the color of large and small circles demonstrating the type of cell and the type virus, which caused the infection. Submodel (C) shows an extended model drawn in (A), where the apoptosis pathway is activated in A3G-augmented cells upon their infection. The deformed blue shapes represent infected cells that have undergone apoptosis. Parameters 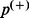 and 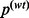 denote the fraction A3G(−) viruses released from infected A3G-augmented and WT cells, respectively. The reduction in the number of released viruses from cells infected by A3G(+) viruses is denoted by *c*. The failure rate of the apoptosis pathway in (C) is represented by *r*. Model I is solely described by submodel (A) while Models IIa, IIb, and IIc consist of submodels in (A) and (B). Model III is comprised of submodels in (B) and (C).

As mentioned earlier, infected A3G-augmented cells release both A3G(−) and A3G(+) viruses. In the model, parameter 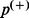 taking values between 0 and 1, denotes the fraction of A3G(−) viruses released from an infected cell and therefore is termed *A3G-free virus release ratio*. The superscript on 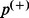 denotes that it is a property of 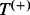. As seen in equations (4d) and (4e), for every *N* viruses that are produced 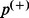 *N* are A3G(−) viruses whereas (1-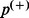)*N* are A3G(+) viruses. The value of 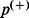 depends on the concentrations of Vif and of A3G expressed in the cell, the kinetics of viral production and release and whether WT or mutated variants of A3G is expressed in the cell. To compute 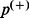, we use our previously built single-cell model [42], where the A3G-Vif interaction along with other intracellular events such as production and degradation of host and viral proteins, and assembly and release of new virions are described using differential equations. The value of 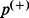 is inversely associated with the intracellular A3G getting encapsulated in the released viral particles, i.e., the higher the production rate of A3G inside the cell, the lower the value of 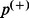. It has been observed that in single-round infectivity assays, cells infected by A3G(+) viruses produce fewer virions compared to cells infected by A3G(−) viruses [10]. Parameter *c* denotes the reduction in the number of virions released from cells infected by A3G(+) viruses, i.e., 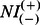 vs. 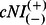 in equations (4d) and (4e). This parameter takes values between 0 and 1. Estimates of *c* have been obtained using computational models of *in vitro* T cell culture infectivity assays [42]. In the model, it is also assumed that cells infected by A3G(−) or A3G(+) viruses have the same death rate. Fig. 3A shows a schematic illustration of Model I. This model provides a benchmark for the optimum performance that A3G-SCT can achieve in modulating *in vivo* HIV replication for given values of 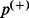 and *c*.

The observed reduction in the number of produced viruses from cells infected by A3G(+) viruses could in fact be explained by decreased infectivity rate of A3G(+) viruses or decreased level of virus production in cells infected by A3G(+) viruses (represented by *c* in Model I) – or both. To compare the effect of decreased infectivity rate of A3G(+) viruses with that of decreased level of virus production in cells infected by A3G(+) viruses, we present a modified version of Model I, where A3G(+) viruses have reduced infectivity (represented by *η* in Model Ib in the supplementary information) compared to A3G(−) viruses. But when infection occurs, unlike Model I, the number of viruses released from cells infected by A3G(+) viruses is the same as that of cells infected by A3G(−) viruses, i.e., all infected cells have the same burst size. As we will see later in the results section, the level of reduction in the reproductive ratio for both Models I and Ib are the same given that the reduction in infectivity rate of A3G(+) viruses is equal to the reduction in level of virus production in cells infected by A3G(+) viruses, i.e., *c* = *η*. This suggests that from a modeling point of view, both of these hypotheses could explain the results of single-round infectivity assays, where cells infected by A3G(+) viruses produce fewer virions compared to cells infected by A3G(−) viruses. However, from a mechanistic point of view, the encapsulated A3G would be unlikely to have effects on viral entry. This is because the entry process involves the binding of gp41 and gp120 on the viral envelope to CD4 and chemokine coreceptors on the T cell surface, whereas during fusion and entry, A3G is inside the viral capsid and does not interact with the proteins on the viral envelope. Therefore, viral entry and infectivity rate constant are assumed to be the same for both types of virus. On the other hand, A3G inhibits several steps during integration and reverse transcription, resulting in production of many nonfunctional viral particles, i.e., reduction in the number of functional viruses released from the cells infected by A3G(+) viruses.

### Model IIa: The Basic HIV Model for WT and A3G-Augmented Cells

We next take into account that it may not be possible to transfect all the CD34+ stem cells and therefore not all the CD4+ T cells would overexpress A3G. Therefore, we extend Model I to include two subpopulations of uninfected cells: A3G-augmented cells that overexpress A3G and WT cells that express A3G at normal levels. Initial infection occurs with a certain amount of A3G(−) viruses. These viruses can infect both WT and A3G-augmented cells. WT CD4+ T cells express A3G at low levels such that Vif can inhibit most of the A3G encapsulation into virions. When WT cells become infected they produce mostly A3G(−) virions and less A3G(+) virions, i.e., 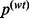 takes high values in the range [0, 1] (Fig. S1 and Table S1 in Method S1, and [89]). In contrast, infected A3G-augmented cells produce a higher fraction of A3G(−) and a lower fraction of A3G(+) virions, i.e., 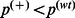. The superscript on *p* denotes whether it is a property of WT or A3G-augmented cells. The newly released A3G(+) viruses will similarly infect both WT and A3G-augmented cells. But these infected cells produce fewer virions. Note that WT cells even after infection by A3G(+) viruses still produce more A3G(−) virions than A3G(+) virions. Model IIa can be defined by a system of eight differential equations.
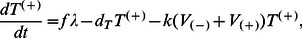
(5a)

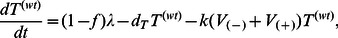
(5b)

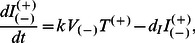
(5c)

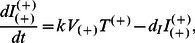
(5d)

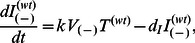
(5e)

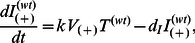
(5f)

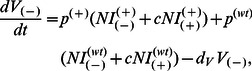
(5g)

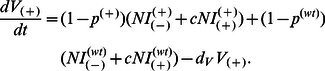
(5h)


Where the concentration of A3G-augmented and WT cells are denoted by 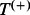 and 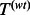, respectively. Parameter *f* represents the fraction of uninfected A3G-augmented cells. In this model there are four subpopulations of infected cells. 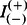 and 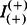 represent the concentration of A3G-augmented cells that are infected by A3G(−) and A3G(+) viruses, respectively. Similarly, WT cells infected by A3G(−) and A3G(+) viruses are represented by 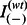 and 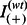, respectively. In general, for *I* variables, superscripts represent whether infected cells are WT or A3G-augmented, while subscripts denote what type of virus is the cause of infection. The infectivity rate constant is assumed to be the same for all virus-cell pairs. All the infected cells have the same death rate. As described, Model IIa has two submodels describing HIV infection in A3G-augmented and WT cells, drawn in Figs. 3A and 3B, respectively. The purpose of this model is to investigate what percentage of the cells must overexpress A3G to block *in vivo* viral replication.

### Model IIb: The Basic HIV Model for WT and A3G-Augmented Cells with Lower Death Rates for Infected A3G-Augmented Cells

As mentioned above, A3G(+) viruses are mostly produced by infected A3G-augmented cells. These viruses are considered to be less harmful than A3G(−) viruses. This is because they cause the infected cells to produce fewer virions than do A3G(−) viruses. Therefore, it can be hypothesized that if infected A3G-augmented cells, as the main source for producing A3G(+) viruses, live longer (die more slowly) compared to infected WT cells, we may achieve a better performance in blocking replication. We test the effect of this possible difference in cell lifespan by customizing A3G-augmented cells to have a lower death rate than WT cells after they get infected. To change the death rate of infected WT and A3G-augmented cells in Model IIa, we only need to replace equations (5c–5f) with equations (6c–6f).
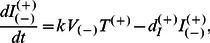
(6c)

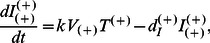
(6d)

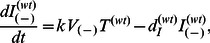
(6e)

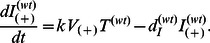
(6f)


Where 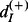 and 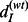 represent the death rate of infected A3G-augmented and WT cells, respectively. Similar to our notations for *I* variables, superscripts on *d* parameters denote whether infected cells whose death rate is represented are WT or A3G-augmented. Note that 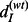 is the same as 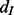 defined in the basic model of HIV infection; however, 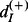 takes smaller values than 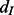.

### Model IIc: The Basic HIV Model for WT and A3G-Augmented Cells with Lower Death Rates for Cells Infected by A3G(+) Viruses

After infection, A3G(+) viruses cause cells to produce and release fewer virions than do A3G(−) viruses. Therefore, these viruses might be considered less toxic for cells and as a result cells infected by A3G(+) viruses might live longer. An important question to ask is therefore, how would the efficacy of A3G-SCT change in a model that has a lower death rate for cells infected by A3G(+) viruses? To find the answer, we replace equations (5c–5f) with equations (7c–7f).
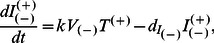
(7c)

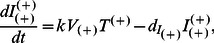
(7d)

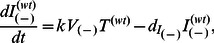
(7e)

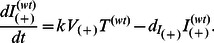
(7f)


Where the death rate of cells infected by A3G(+) and A3G(−) viruses are denoted by 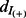 and 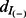, respectively. Similar to our notations for *I* variables, subscripts on *d* parameters denote whether cells whose death rate is represented were infected by A3G(−) or A3G(+) viruses. Note that 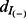 is the same as 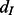 defined in the basic model of HIV infection; however, 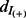 takes smaller values than 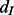.

### Model III: The Basic HIV Model for WT and A3G-Augmented Cells with Auto-Apoptosis Capability

For stem cell transfection, the effector gene, in this study a WT A3G or a functional A3G variant, can be included in a conditional gene circuit with an appropriate biosensor (as opposed to under the control of a constitutive promoter). One such biosensor is the HIV LTR promoter: the HIV protein Tat binds to the promoter and initiates expression of the effector gene [90]–[92]. In this way, transfected A3G is unexpressed until virus entry is detected, and only then is anti-HIV protein expression boosted. This provides on-demand antiviral activity at the cellular level with low potential for side effects. Other functionalities can also be added to the circuit to make it more potent against HIV infection. For example, an additional effector gene can be added to induce activation of the apoptosis pathway upon infection, causing the infected cell to die, significantly reducing viral production. Model III as described by the following differential equations is an extension of Model IIa to study the effects of such apoptotic functionality on modulating viral production.
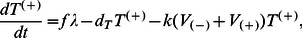
(8a)

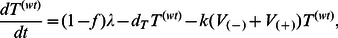
(8b)

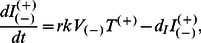
(8c)

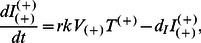
(8d)

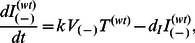
(8e)

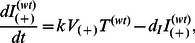
(8f)

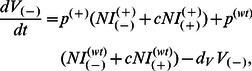
(8g)

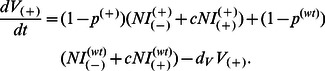
(8h)


A3G-augmented cells with apoptosis capability are assumed to die after infection without producing infectious virions. However, it is also considered that some inefficiencies may be involved with this process, suggesting that some A3G-augmented cells can survive after infection. This is captured by the inefficacy rate *r* in equations (8c) and (8d), where *r* is the fraction of infected A3G-augmented cells that have escaped auto-apoptosis. Model III submodels are visualized in Figs. 3B and 3C.

## Results

**Figure 4:**
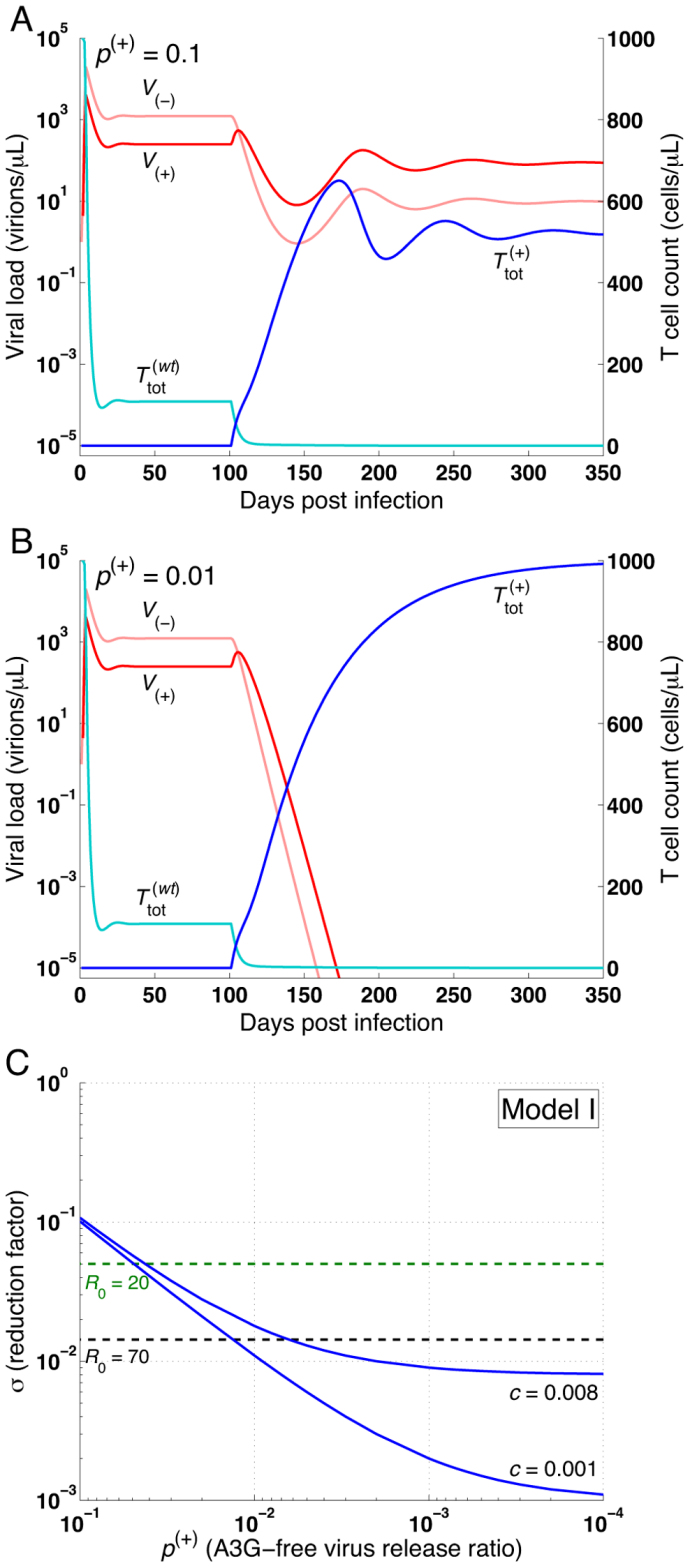
Effects of A3G-free virus release ratio on HIV replication and reproductive ratio in Model I. Infection occurs on day 0 and A3G-SCT begins on day 100 (Model I assumes that all the cells overexpress A3G). The impact of the therapy on the total concentration of viruses and cells is shown for (A) 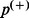 = 0.1 and (B) 0.01. Light and dark red lines represent 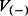 and 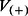 variables, respectively, while light and dark blue lines represent 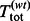 and 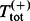 variables, respectively. The left and right axes show the virus and cell concentrations. Parameter *c* is given the value of 0.001. Although (A) 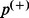 = 0.1 decreases the viral load and increases the T cell count, it cannot eradicate the virus (*R*_1_ = 2.02). However, (B) 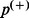 = 0.01 successfully reduces *R*_1_ to 0.22 and the infection dies out. (C) shows the level of reduction in the reproductive ratio that can be achieved by A3G-SCT for different values of 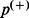 and *c*. The green and black dashed lines represent the minimum level of reduction needed to stop HIV replication for *R*_0_ = 20 and 70, respectively. Note that from left to right on the bottom axis, the A3G-free virus release ratio decreases from 10^−1^ to 10^−4^. Simulation results suggest that for small values of 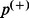 and *c*, up to three orders of magnitude reduction can be achieved.

**Figure 5:**
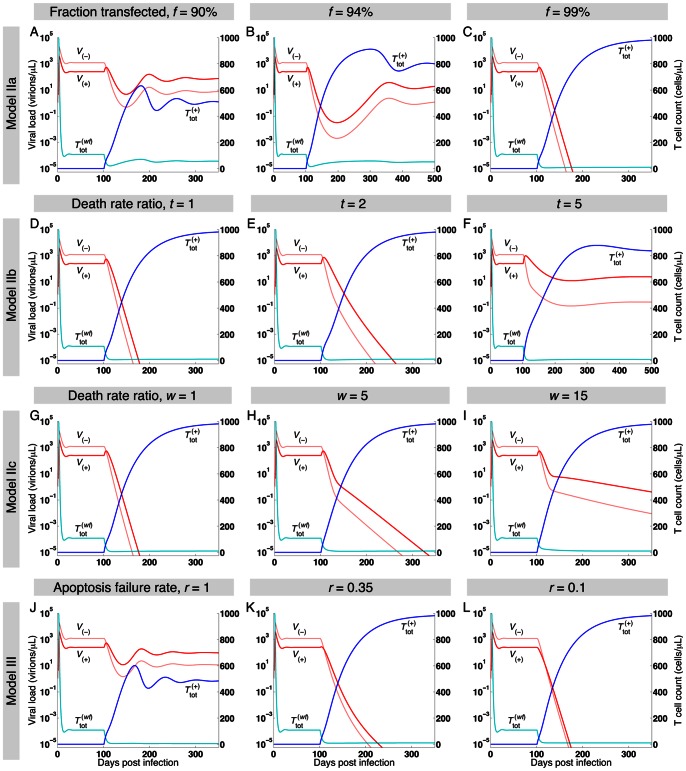
Effects of percentage of transfected cells, death rate ratios, and auto-apoptosis failure rate on HIV replication in Models IIa, IIb, IIc, and III. In all simulations, infection occurs on day 0 and A3G-SCT begins on day 100. In all the subfigures, light and dark red lines represent 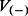 and 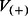 variables, respectively, while light and dark blue lines represent 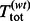 and 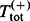 variables, respectively. The left and right axes show the virus and cell concentrations. Parameter *c* is given the value of 0.001. *f* = 99% for all the subfigures except (A) and (B) where *f* = 90% and 94%, respectively. For the first three rows, 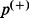 = 0.01 (Models IIa, IIb, and IIc), while it is set to 0.1 for the last row (Model III). (A–C, *R*_2a_ = 1.86, 1.2, and 0.38) simulation results for Model IIa suggest that high percentage of A3G-augmented cells is required to stop *in vivo* HIV replication. (D–F, *R*_2b_ = 0.38, 0.6, and 1.25) Model IIb assumes lower death rates for infected A3G-augmented cells compared to infected WT cells, i.e., *t* >1. Simulation results suggest that the efficacy of the therapy is degraded as the value of *t* increases. (G–I, *R*_2c_ = 0.38, 0.46, and 0.66) A3G(+) viruses are assumed to be less toxic in Model IIc. Therefore, cells infected by these viruses die more slowly compared to cells infected by A3G(−) viruses, i.e., *w* >1. Model IIc predicts that lower death rates for cells infected by A3G(+) viruses have a diminishing effect on the performance of the therapy. (J–L, *R*_3_ = 2.16, 0.87, and 0.37) In Model III, cells are equipped with an additional gene circuit that activates apoptosis pathway upon infection; however, the circuit has a failure rate of *r*. Simulation results indicate that providing cells with this additional gene circuit enhances the performance of the therapy and can reduce the reproductive ratio to values less than one even in cases that the A3G-free virus release ratio does not take very small values.

In all the simulations, the initial concentration of uninfected WT CD4+ T cells is 1000 cells/µl. Without loss of generality, the concentrations of A3G(+) and A3G(−) viruses are given initial values of zero and one virions/µl, respectively. A3G-augmented stem cell therapy (A3G-SCT) is introduced on day 100, long after the system has reached the steady state. *R*_0_ = 20 in our simulations (Table 1). However, in figures, we also plot the level of reduction required to block *in vivo* HIV replication with *R*_0_ = 70, a conservatively high value for the reproductive ratio that might occur temporarily during the course of infection. This shows the performance of A3G-SCT in the worst-case scenarios. In Figs. 4A–B and 5A–L, 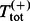 refers to the total concentration of uninfected and infected A3G-augmented cells, i.e., 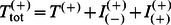 in Models I, II, and III. Analogously, in Models II and III, 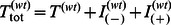, while in Model I, 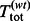 refers to the total concentration of uninfected and infected WT CD4+ T cells before introducing the therapy. We use 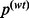 = 0.83 in our simulations throughout this paper. The value of 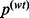 was estimated using our previously published model [42] and WT HIV replication data in A3G-expressing and A3G-knockout cells [89] (Method S1).

### A3G Gene Therapy can Effectively Stop *in vivo* HIV Replication

Analyzing Model I at steady state and assuming that the virus grows and establishes infection, we can derive the reproductive ratio, given by the following equation.
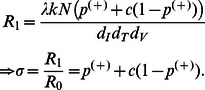
(9)


Where *σ* is the reduction factor. Detailed derivation of equation (9) can be found in Method S2. Since both 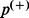 and *c* take values between zero and one, the reproductive ratio for Model I is reduced compared to the reproductive ratio for the basic model of HIV infection. With 100% of stem cells transfected with A3G and 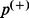 = 0.1 (90% of the budded virions from each cell carry A3G), the therapy fails to reduce *R*_1_ to values less than one (*R*_1_ = 2.02), however, it causes a 4.8-fold increase in the concentration of T cells and a 15.3-fold decrease in the total concentration of viruses (Fig. 4A). By decreasing 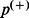, the A3G-free virus release ratio, to 0.01 (increasing the percentage of budded virions carrying A3G to 99%), the therapy successfully eradicates the virus from the body and the concentration of CD4+ T cells goes back to the initial concentration of 1000 cells/µl (Fig. 4B, *R*_1_ = 0.22).

Fig. 4C shows that A3G-SCT can reduce the basic reproductive ratio by three orders of magnitude for small values of 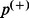 and *c*. However, in order to reduce the reproductive ratio of 20 and 70 to less than one, 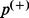 only needs to take values less than 0.049 and 0.013, respectively when *c* = 0.001, suggesting the potential of A3G-SCT to successfully achieve a functional cure for HIV infection. Note that parameter *c* plays a limiting role in reducing the reproductive ratio such that as 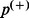 goes to zero, the maximum amount of reduction that can be achieved is 1/*c* fold. Depicted curves in Fig. 4C represent the best performance that can be achieved with this treatment for given values of 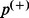 and *c*.

The modified version of Model I assumes that A3G(+) viruses have reduced infectivity (represented by *η* in Model Ib in Method S3) compared to A3G(−) viruses. But, the number of viruses released from cells infected by A3G(+) viruses is the same as that of cells infected by A3G(-) viruses. The reproductive ratio for Model Ib is exactly the same as equation (9) if *c* is replaced with *η* (compare equation (9) with equation (SIb-9) in Method S3). This suggests that reduction in infectivity rate of A3G(+) viruses has the same effect on the reproductive ratio as does the reduction in level of virus production in cells infected by A3G(+) viruses. However, the later is more relevant from a mechanistic point of view.

### Blocking Replication Requires High Percentage of A3G-Augmented Cells

Since complete stem cell transfection may not be feasible, Model IIa splits CD4+ T cells into two subpopulations of WT and A3G-augmented cells to evaluate the limitations of imperfect transfection. Studying the model at steady state (Method S4), we find that the reproductive ratio can be written by
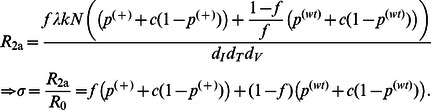
(10)


*R*_2a_ is reduced compared to the basic reproductive ratio because parameters 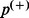, 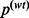, *c*, and *f* take values less than one. However, it takes values greater than *R*_1_ for *f* <1, suggesting that imperfect transfection reduces the efficacy of A3G-SCT. The reduction factor, *σ*, shown in equation (10) can be intuitively calculated from the reduction factor obtained in Model I. This is because now there exists two cell subpopulations in the system: A3G-augmented cells with the reduction factor equal to (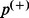 *+c*(1−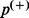)); and WT cells with *σ* = (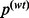 *+c*(1−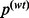)). The total reduction factor is hence the sum of the reduction factor for each cell subpopulation multiplied by its fraction in the total population. Given that 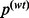 takes a relatively high value of 0.83, one should note that the reduction caused by WT cells in the reproductive ratio has a lesser impact than that of A3G-augmented cells.

Although A3G-SCT with *f* = 90% (*R*_2a_ = 1.86) and 94% (*R*_2a_ = 1.20) causes 18.2- and 77-fold reduction in viral load and improves total T cell concentration by 5.2- and 7.8-fold, respectively, the virus is not eradicated and the infection is still spread in the body (Figs. 5A and 5B, 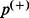 = 0.01). Increasing the percentage of A3G-augmented cells to 99% reduces *R*_2a_ to 0.38 and causes the HIV infection to die out (Fig. 5C, 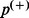 = 0.01).

**Figure 6:**
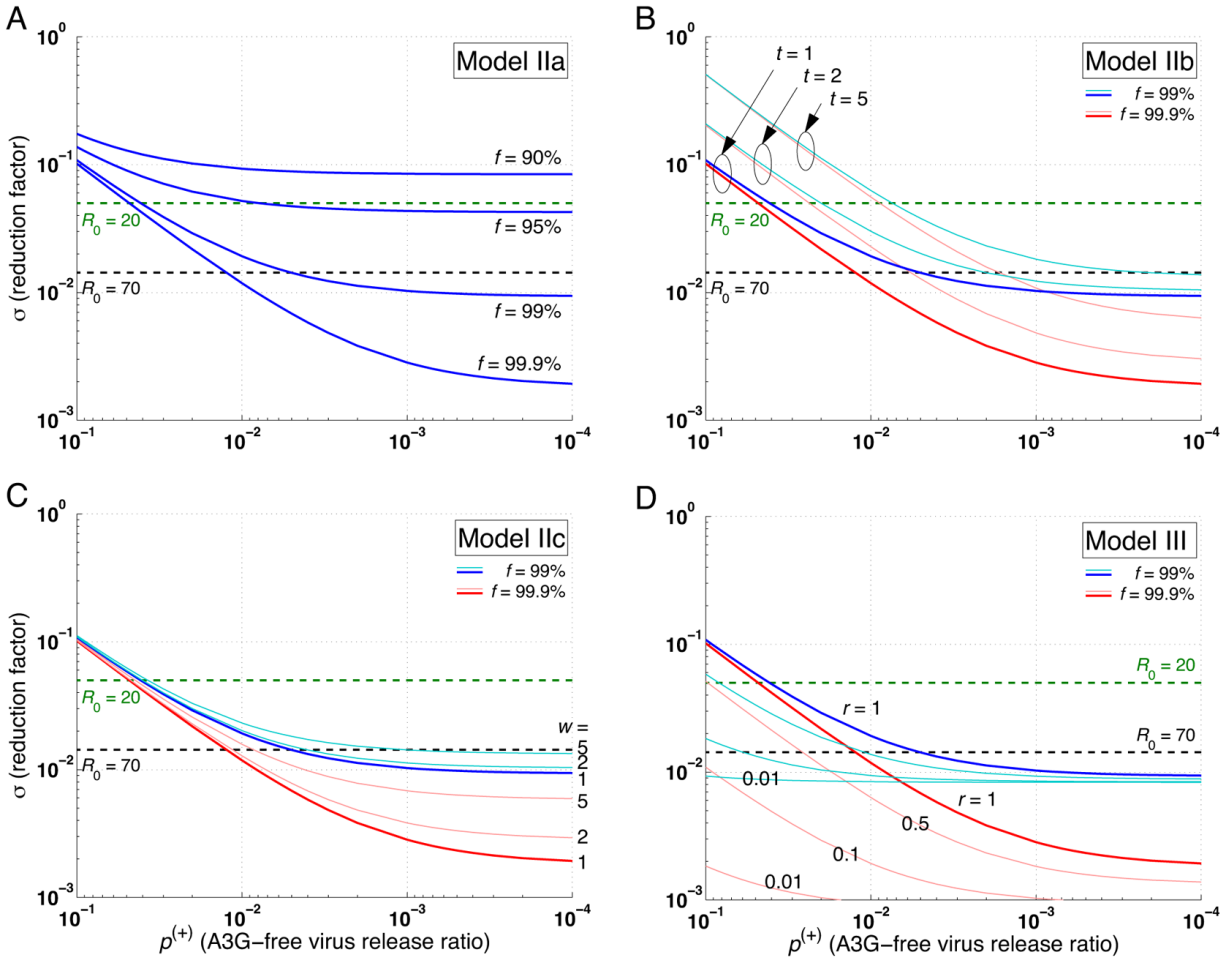
Effects of percentage of transfected cells, death rate ratios, and auto-apoptosis failure rate on reproductive ratio in Models IIa, IIb, IIc, and III. The level of reduction in the reproductive ratio that can be achieved by A3G-SCT for different values of 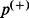 is shown for each model. In all the subfigures, the green and black dashed lines represent the minimum level of reduction needed to stop HIV replication for *R*_0_ = 20 and 70, respectively. Note that from left to right on the bottom axis, the A3G-free virus release ratio decreases from 10^−1^ to 10^−4^. Parameter *c* is given the value of 0.001. (A) Model IIa suggests that *f* = 95% is required to block HIV replication for *R*_0_ = 20. Higher values of *f* are needed to block HIV replication for larger values of *R*_0_. (B) Simulation results of Model IIb predict that the performance of the therapy will be degraded if infected A3G-augmented cells die more slowly compared to infected WT cells, i.e., when *t* >1. (C) Model IIc also suggests that the therapy achieves lower efficacy if cells infected by A3G(+) viruses die more slowly than cells infected by A3G(−) viruses, i.e., when *w* >1. However, the performance degradation is less severe than that of Model IIb. (D) Finally, Model III indicates that A3G-SCT can achieve better efficacy if infected A3G-augmented cells activate apoptosis pathway upon their infection.

Reduction in the reproductive ratio in Model IIa is shown for several values of *f* in Fig. 6A. For *f = *95–99%, A3G stem cell transfection can reduce the reproductive ratio by a factor of 23.5 to 107.6. Note that as 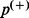 and *c* go to zero, the fold reduction in the reproductive ratio approaches 1/(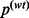
(1−*f*)). Therefore regardless of how small 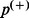 and *c* values are, the percentage of WT cells will determine the performance of the treatment. Fig. 6A also shows that large reductions in the reproductive ratio can be achieved as *f* approaches 1.

### Lower Efficacy is Achieved if Infected A3G-Augmented Cells Die More Slowly than do Infected WT Cells

Model IIb describes a special case when A3G-augmented CD4+ T cells can live longer after they get infected, resulting in lower death rates for these cells compared to infected WT cells. Calculating the reproductive ratio for Model IIb illustrates whether this strategy is beneficial. Modification of the death rate of infected A3G-augmented cells results in a minor change in equation (10). The new reproductive ratio can be written as (Method S5)
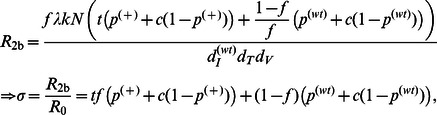
(11)where 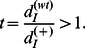


Based on our assumption, *t* must take values greater than one, which suggests lower therapeutic performance if infected A3G-augmented cells live longer than infected WT cells, i.e., *R*_2b_>*R*_2a_ for *t* >1.

Figs. 5D–F show that as the value of *t* increases, the performance of A3G-SCT diminishes (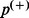 = 0.01, *f* = 99%). For *t* = 2, the A3G treatment can still eradicate the virus from the body (Fig. 5E, *R*_2b_ = 0.60), but the rate of decline in viral load is slower than that of *t* = 1 (Fig. 5D, *R*_2b_ = 0.38). Nonetheless, eradication is not possible for *t* = 5 (Fig. 5F, *R*_2b_ = 1.25).

As seen in Fig. 6B, the highest reduction in the reproductive ratio is achieved when *t* = 1, i.e., when infected A3G-augmented cells have the same death rate as infected WT cells. Also as 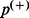 decreases, the gap between the performance of A3G-SCT with *t* = 1 and that of therapy with *t*>1 decreases, meaning that for small values of 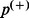, the importance of difference between death rate of infected A3G-augmented and WT cells is less significant.

### Lower Efficacy is Achieved if Cells Infected by A3G(+) Viruses Die More Slowly than do Cells Infected by A3G(−) Viruses

Model IIc focuses on a scenario where A3G(+) viruses are assumed to be less toxic. This is because they cause the infected cells to produce fewer virions than do A3G(−) viruses. Therefore, cells infected by A3G(+) viruses may live longer after getting infected, i.e., they have a lower death rate than that of cells infected by A3G(−) viruses. By calculating the equilibrium point for Model IIc in Method S6, the reproductive ratio is obtained by
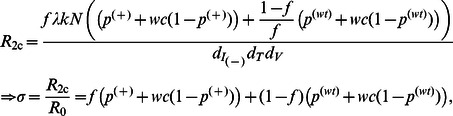
(12)where 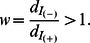


Since *w* is assumed to be greater than one, the performance of A3G-SCT is degraded compared to Model IIa (*R*_2c_>*R*_2a_ for *w* >1). However, the effect of *w* on the performance of A3G-SCT is less severe than that of *t* in Model IIb presented in the previous section; for *f* ≥50%, *c* = 0.001 and *t* = *w* >1, we always have *R*_2c_<*R*_2b_, unless 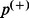 >1.7×10^−4^. This can be seen by comparing Figs. 6B and 6C. In order to reduce the reproductive ratio from 70 to less than one with *f* = 99.9%, the A3G-free virus release ratio, needs to be decreased by 1.1 or 1.5-fold when *w* goes from 1 to 2 or 5, respectively (Fig. 6C). This shows that *w* has a mild effect on the performance. In contrast, these numbers are 2.2- and 7.4-fold when *t* goes from 1 to 2 or 5, respectively (Fig. 6B).

Comparing Figs. 5G–I, we observe that the efficacy of A3G-SCT decreases as the value of *w* increases (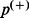 = 0.01, *f* = 99%). For *w* = 5 (Fig. 5H, *R*_2c_ = 0.46) and *w* = 15 (Fig. 5I, *R*_2c_ = 0.66), the therapy can eradicate the virus from the body but the rate of decline in viral load is slower than when *w* = 1 (Fig. 5G, *R*_2c_ = 0.38). By comparing Figs. 5H and 5F, it can be seen that for the same values of *w* and *t*, the rate of decline in virus concentration is faster, the T cell count is higher, and the viral load is lower in Model IIc compared to Model IIb.

### A3G-Augmented Cells with Auto-Apoptosis Capability Can Stop Replication more Efficiently

Finally, Model III explores the possibility of enhancing efficacy of the treatment by co-transfecting stem cells with A3G and a gene circuit that induces activation of the apoptosis pathway in progeny CD4+ T cells upon infection by HIV. The gene circuit causes the cell to die after infection and hence it cannot produce any infectious virions. However, if the infected cell somehow escapes the apoptosis pathway, then A3G overexpressed in that cell gets encapsulated into virions to induce its antiviral activities. The reproductive ratio for Model III is given by (Method S7)
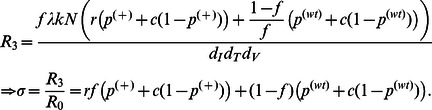
(13)


Since *r* takes values less than one, the reproductive ratio of Model III is reduced compared to that of Model IIa, suggesting that a better efficacy can be achieved using co-transfection of stem cells with A3G and an auto-apoptosis gene circuit. Reduction in the reproductive ratio for several values or *r* is shown in Fig. 6D. For *f = *99% and *r = *0.1, A3G-SCT reduces the reproductive ratio of 70 to less than one for any value of 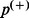 less than 0.059. For *r* = 0.01, 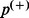 should only take values less than 0.60 to be able to eradicate the virus from the body. This greatly relaxes the pressure on parameter 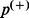, the A3G-free virus release ratio, to take values as small as 0.005 for *r* = 1 to eradicate the virus, suggesting that using this therapy A3G or its variants can be overexpressed at much lower concentrations, and still be effective in blocking HIV replication. Note that the improvement in the performance of therapy becomes less significant for small values of 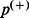 (Fig. 6D).

For *f* = 99% and 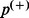 = 0.1, when *r* = 1, i.e., the apoptosis gene circuit fails all the time, the treatment reduces the viral load but cannot eradicate the virus from the body (Fig. 5J, *R*_3_ = 2.16). As *r* decreases to 0.35 (the apoptosis gene circuit works 65% of the time), the treatment can successfully eradicate the virus and the infection goes away (Fig. 5K, *R*_3_ = 0.87). For a smaller value of *r* in Fig. 5L while *f* = 99% and 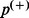 = 0.1 (*R*_3_ = 0.37), the rate of decline in viral load is even faster than the case of *f* = 99% and 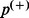 = 0.01 in Fig. 5C (*R*_1_ = 0.38).

### A3G^ΔVif^ Outperforms A3G in Blocking *in vivo* HIV Replication

Using the results of our previously published single-cell model [42], we study the effects of A3G and A3G^ΔVif^ (an A3G variant that does not bind Vif such as A3G/F126–129 [43] and D128KA3G [44]) overexpression on the reproductive ratio.

**Figure 7:**
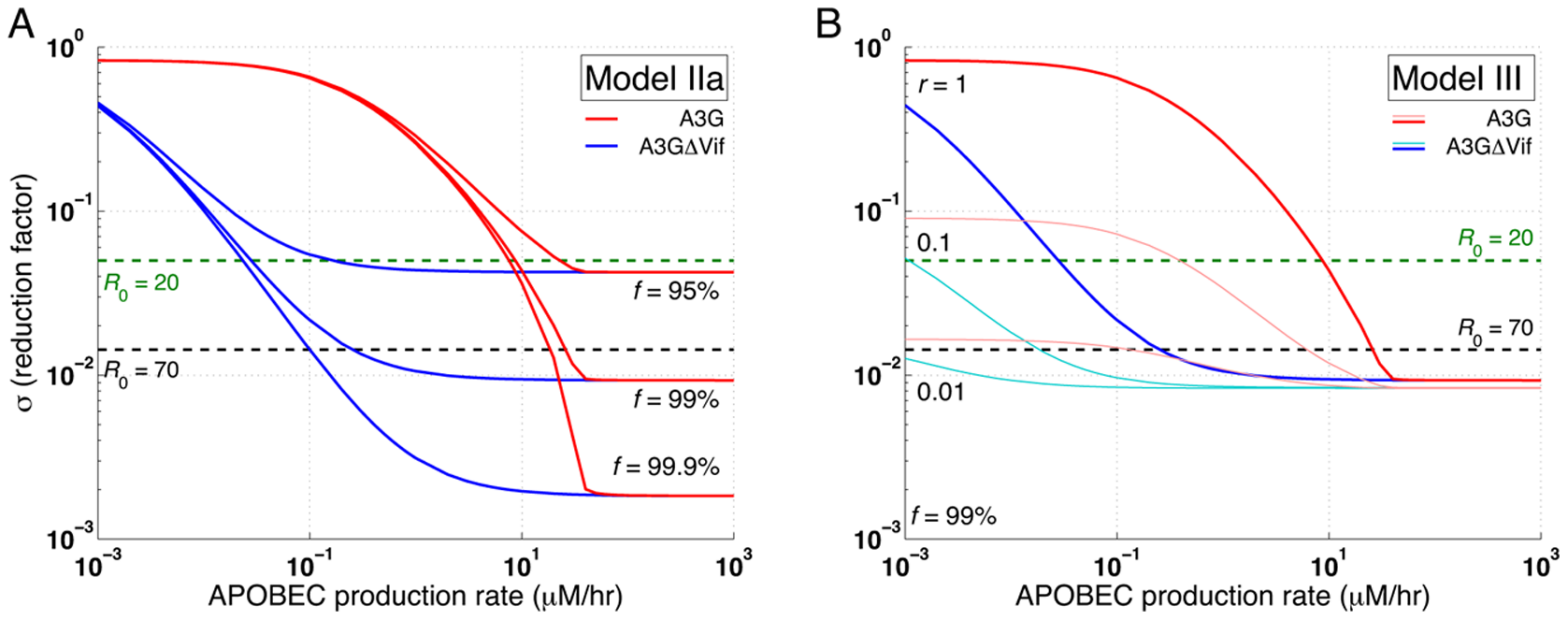
Effects of A3G and A3G^ΔVif^ overexpression on reproductive ratio in Models IIa and III. The level of reduction in the reproductive ratio that can be achieved by overexpression of A3G (red) and A3G^ΔVif^ (blue) is shown. In the two subfigures, the green and black dashed lines represent the minimum level of reduction needed to stop HIV replication for *R*_0_ = 20 and 70, respectively. Parameter *c* is given the value of 0.001. (A) Simulation results for Model IIa show that almost two orders of magnitude lower production of A3G^ΔVif^ compared to that of A3G is required to achieve the same performance. (B) Model III predicts that by decreasing the apoptosis failure rate, lower production rate of A3G and A3G^ΔVif^ is required to stop *in vivo* HIV replication.

Fig. 7A shows the reduction in the reproductive ratio achieved in Model IIa versus the production rate of A3G and A3G^ΔVif^. The APOBEC production rate in Fig. 7 is in addition to the normal level of A3G production in WT cells. For *f* = 99%, in order to reduce the reproductive ratio of 70 to values less than one, the production rate of A3G should be at least 26 µM/hr while this number is 0.252 µM/hr for A3G^ΔVif^. Analogously, a large gap is seen between red (A3G) and blue (A3G^ΔVif^) curves for other values of *f*, suggesting that nearly two orders of magnitude lower production rate of A3G^ΔVif^ is required to achieve the same efficacy as that of A3G. Simulation results for Model III show that as *r* decreases, the gap between red (A3G) and blue (A3G^ΔVif^) curves widens (Fig. 7B, *f* = 99%). For *r* = 1, in order to reduce the reproductive ratio of 70 to one, A3G^ΔVif^ production rate is 103-fold lower than that of A3G; this number is 331-fold lower for *r* = 0.01. Note that for all the curves in both Figs. 7A and 7B, the reduction factor does not decrease further after a certain production rate. This is because the percentage of WT cells, 1−*f*, determines the maximum achievable performance of the treatment, regardless of how much A3G or A3G^ΔVif^ is overexpressed.

### Impact of Mixed Levels of A3G Overexpression on the Performance of A3G-SCT

In Models I, II and III, it is assumed that cells are either WT (expressing A3G at normal levels) or A3G-augmented (overexpressing A3G at high levels). However, stem cell transfection is not an all-or-none phenomenon, i.e., after the transfection, some of the progeny CD4+ T cells overexpress A3G at high levels while others may express A3G at lower levels. Therefore, it is noteworthy to evaluate the performance of A3G-SCT when all CD4+ T cells express A3G higher than WT cells, but the overexpression can be either low or high. Model IV (defined in Method S8) gives insights into how the performance of the therapy would change in this scenario. 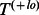 and 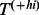 represent two subpopulations of cells overexpressing A3G at low and high levels, respectively. We assume that 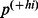
<
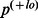
<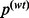. Similar to Model IIa, the reproductive ratio for Model IV can be mathematically derived as (Method S8)
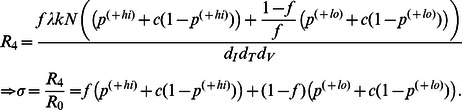
(14)


**Figure 8:**
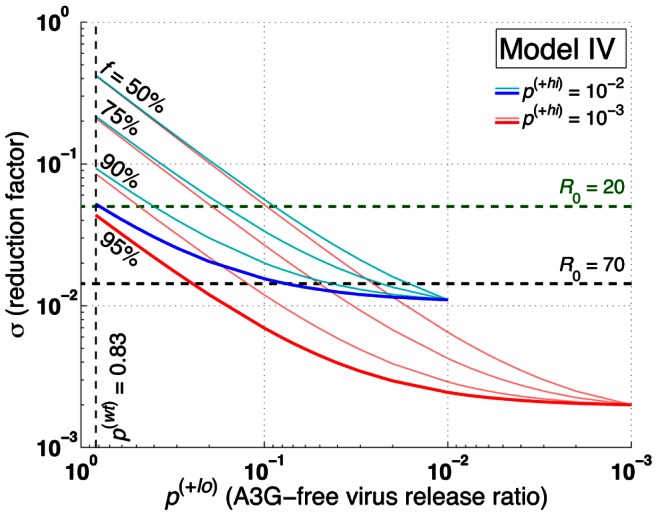
Effects of 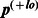, A3G-free virus release ratio, on reproductive ratio. The level of reduction in the reproductive ratio that can be achieved by A3G-SCT for different values of 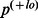 is shown when 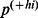 = 0.001 (red) and 0.01 (blue). In our simulations, 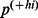
<
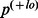
<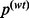. The green and black horizontal dashed lines represent the minimum level of reduction needed to stop HIV replication for *R*_0_ = 20 and 70, respectively. Note that from left to right on the bottom axis, 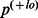 decreases from 0.83 to 10^−3^. Parameter *c* is given the value of 0.001.

As seen in Fig. 8, for *f* = 90% and 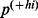 = 0.001, the other 10% of the cells that overexpress A3G at low levels need to have 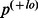 taking values less than 0.48 in order for A3G-SCT to reduce *R*_0_ from 20 to one, i.e., only 52% of the viruses released from these cells must carry A3G for a successful therapy. Note that 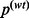 = 0.83, hence 17% of viruses released from WT infected cells are already A3G(+). To achieve 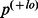 = 0.48, production rate of transfected A3G can be 119-fold lower than that of cells expressing A3G at high levels. Increasing 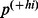 to 0.01 while keeping *f* unchanged results in 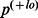 = 0.40 to have a reduction factor equal to 20. For 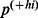 = 0.01, when *f* decreases to 50%, i.e., the maximum achievable reduction in the reproductive ratio is only 2.4-fold in Model IIa, the therapy can cause 20-fold reduction only if 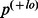 takes values less than 0.09 (Fig. 8).

## Discussion

Despite decades of research, 33 million people live with HIV; 2.6 million people are infected annually, with 1.8 million fatalities [93]. Current leading-edge treatment regimens such as highly active antiretroviral therapy (HAART) ensure that millions of people with HIV lead normal lives, and prevent millions of additional infections by reducing infectivity. However, HAART is expensive, is not a cure, and only a fraction of HIV sufferers worldwide receive it. There remains a critical need for new therapies, especially cures. Cures are preferable because they can eliminate long-term maintenance costs and issues of adherence to long-term regimens.

The idea of gene therapy and stem cell transfection has recently renewed hope to achieve a functional cure for HIV. The reported cure of the “Berlin Patient” was achieved through transplantation of hematopoietic stem cells from a donor with two key characteristics: (a) donor was tissue matched for transplantation; (b) donor had a genetic mutation that conferred resistance to HIV [69]–[71]. Finding such a donor would be difficult in general, but matched donor stem cells could be augmented, to provide the HIV-resistance by inserting genes or gene networks into those cells before transplantation. Small molecules (such as HAART drugs) cannot be encoded, but overexpression of endogenous anti-HIV proteins such as APOBEC3G, which can efficiently inhibit viral reproduction, is possible; alternatively or in addition, encoding a pro-apoptotic stimulus would induce HIV-infected CD4+ T cells to die, shortening lifespan and limiting HIV production. Gene therapy has the potential to counter problems associated with current anti-HIV treatments such as drug side effects, patient adherence, and emergence of drug resistant viruses. For some patients, multiple rounds of low adherence and viral resistance can lead to exhaustion of all antiviral regiments. Gene therapy can be promising in these cases; however, in order for it to become a standard of care in treating HIV, issues such as safety and persistence of genetically modified cells in the body must be addressed.

Here, we developed mathematical models extending the basic model of *in vivo* HIV infection to describe the impacts that delivery of stem cells transfected with wild type A3G or its variants can have on HIV replication. A key novel feature of this work is the incorporation of previously developed biologically validated model of A3G-Vif interactions in a single cell [42] into the established model of *in vivo* HIV infection [73]–[78]. Our models can be generalized to describe and simulate other anti-HIV therapies. Two crucial parameters of these models are *p* and *c*. In the general form, parameter *p* is the fraction of released viruses from a single cell that are unaffected by a drug, an anti-HIV protein, or a restriction factor. On the other hand, parameter *c* is the reduction in the number of viruses released from cells infected by affected viruses. Knowing *p* and *c* for a restriction factor, we can calculate the efficacy of the therapy.

In our simulations, A3G-augmented stem cell therapy (A3G-SCT) is introduced on day 100 after initial infection by changing the production of uninfected CD4+ T cells in thymus to generate both WT and A3G-augmented CD4+ T cells with the ratio of 1-*f*:*f*. In reality, for stem-cell based gene therapy, CD34+ stem cells would be mobilized using granulocyte colony stimulating factor (GCSF) and harvested from the blood for *ex vivo* gene modifications. Patients may or may not undergo myeloablative conditioning. This procedure is performed prior to bone marrow transplantation using chemotherapy or total body irradiation with the purpose of killing all the stem cells and suppressing the immune response. This in turn leaves the body prone to infections but reduces the risk of graft-versus-host disease. Finally, transfected stem cells are reinfused back into patients. After transplantation, these cells differentiate into cell types such as CD4+ T cells and macrophages that are now able to overexpress anti-HIV genes. All these would result in temporary changes in the values of the system parameters such as production and death rates of CD4+ T cells. However, several weeks after bone marrow transplantation, stem cells and their progenies are sufficiently expanded to restore the immune system and thus we assume that the system parameters take on values similar to those pre-therapy. Therefore, in this paper, we focus on the steady state response rather than the transient response immediately after A3G-SCT to study the impact of therapy on modulating *in vivo* HIV replication.

Model I assumes that all CD34+ stem cells in the body are transfected with A3G or one of its variants. The results for this model demonstrate that A3G-SCT can reduce the reproductive ratio to values less than one for sufficiently small values of 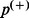, i.e., the concentration of A3G or its variants in HIV-producing cells must be high enough such that it gets encapsulated in most viruses. In the next model, it is assumed that a fraction of stem cells remain untransfected. Model IIa describes the viral dynamics in a mixed population of A3G-augmented and WT CD4+ T cells and predicts that the percentage of stem cells transfected with A3G or its variants must be 95% or higher for the therapy to be able to successfully stop *in vivo* HIV replication when the pre-therapy reproductive ratio is 20. This critical result suggests that A3G-SCT can be an effective functional cure for HIV infection and would provide successful results in most clinical settings where *R*_0_ takes values in the range of 10 to 20 [86]–[88]. Note that the value of *f* can be affected by two major factors: efficiency of stem cell transfection with A3G or its variants; and performance of bone marrow transplantation. Viral vectors can achieve high transfection efficiencies but they raise safety concerns. On the other hand, non-viral delivery systems such as biomaterials are considered to be safe but further research and optimizations are needed to improve their efficiencies. Selection of cells transfected *ex vivo* should lead to high incorporation. In terms of bone marrow transplantation, multiple rounds of reinfusion of CD34+ stem cells may be required in order to establish a high ratio of stem cells that overexpress A3G. This is crucial because the maximum achievable reduction by A3G-SCT is limited by the fraction of WT CD4+ T cells in the body.

Model IIb describes a hypothetical scenario, where infected A3G-augmented cells die more slowly (while the number of released viruses from infected cells per day remains unchanged) compared to infected WT cells. One might expect that the extra condition on infected A3G-augmented cells could change the balance of A3G(−) and A3G(+) viruses at steady state such that the reproductive ratio would be reduced more efficiently. However, the model predicts that having infected A3G-augmented cells live longer leads to lower performances. This unexpected result can be explained if we look at the two subpopulations of target cells. Since there is no interaction/feedback between the two subpopulations, WT and A3G-augmented cells appear to act as isolated subsystems. Therefore, the kinetics of WT subpopulation of cells is untouched, while infected A3G-augmented cells live longer and produce more virions compared to the previous case represented in Model IIa, resulting in a lower decrease in the reproductive ratio.

As mentioned, A3G(+) viruses are considered to be less harmful than A3G(−) viruses. Model IIc predicts that the performance of the therapy will be diminished if cells infected by A3G(+) viruses have a lower death rate than that of cells infected by A3G(−) viruses. Similar to Model IIb, in order to understand this result, we have to take into account that although cells infected by A3G(−) viruses are unchanged, other cells infected by A3G(+) viruses live longer and release more virions. Therefore, the reduction in the reproductive ratio is lower compared to Model IIa, resulting in a lower performance. In the literature, we have not found *in vitro* measurements of death rates for cells infected by A3G(+) viruses. Therefore further studies can clarify whether cells infected by A3G(+) viruses indeed live longer, though the model suggests that the impact of lower death rates for cells infected by A3G(+) viruses is not severe (Fig. 6C).

Effective gene therapy against HIV will likely require a combination of anti-HIV mechanisms. Therefore, in addition to transfecting stem cells with A3G, other gene circuits can also be employed that may improve the performance of the therapy. Model III focuses on a case where stem cells are co-transfected with A3G and an auto-apoptosis gene circuit. Simulation results of Model III demonstrate that the addition of the auto-apoptosis gene circuit eases the requirement to have very small values for 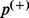 to achieve efficacy. This is an important result because it shows that the auto-apoptosis gene circuit can be beneficial in cases where overexpression of A3G or its variants is toxic or not desirable for some reasons. Model IV relaxes the assumption of all-or none transfection and studies the performance of A3G-SCT when all stem cells are transfected but progeny CD4+ T cells overexpress A3G at varying levels. The model predicts that the performance of A3G-SCT can be significantly improved if cells that overexpress A3G at low levels have somewhat higher A3G production rates than WT cells.

In our model, parameters 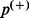 and 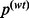, termed A3G-free virus release ratio, create an interface between the previously built single-cell model of A3G-Vif interactions [42] and the *in vivo* models of HIV infection described in this work. The A3G-free virus release ratio is dose-dependent and directly measures the capability of WT A3G or its variants to get encapsulated into released virions. Therefore, it effectively avoids the necessity of taking into account the concentration of anti-HIV proteins in building the *in vivo* models. Having simplified the model, the A3G-free virus release ratio does not shed light on how much protein production is needed to achieve the required reduction in the reproductive ratio. Therefore, integrating the single-cell model results into the *in vivo* models of HIV infection helps us evaluate the required production rate of A3G and A3G^ΔVif^ (a vif resistant A3G by a single amino acid mutation [43], [44]) to stop HIV replication. Simulation results suggest that the production rate of A3G^ΔVif^ can be nearly two orders of magnitude lower than that of A3G to achieve the same performance, suggesting that A3G^ΔVif^ has great potential to be used in gene therapy [72]. This can be useful in cases where overexpression of A3G is toxic to cells. Other therapeutic approaches such as using high-affinity antibodies that bind to Vif [94] or blocking Vif dimerization by small peptides [95] have been suggested in the literature. One should note that both of these approaches attempt to inhibit Vif binding to A3G and hence maximize the efficacy of A3G encapsulation into virions. But the impact of these strategies is limited by the amount of A3G that is expressed in cells. In other words, these molecules cannot reduce the A3G-free virus release ratio sufficiently unless A3G is also overexpressed. In addition, they would not have any effect on the performance of A3G^ΔVif^, because this protein is a variant of A3G that does not bind to Vif in the first place. One potential obstacle in using A3G^ΔVif^ against HIV infection is the high mutation rate of the virus. HIV evolves rapidly and it could mutate Vif protein to restore its ability to bind A3G^ΔVif^ and to suppress anti-HIV activities of this restriction factor. In order to address this issue, similar to the rationale of HAART, other anti-HIV genes should be combined with A3G^ΔVif^ to provide several independent layers of protection against HIV infection, and hence reduce the chance of viral escape [72].

Although more studies need to be done on *in vivo* antiviral effects of A3G, our models suggest that A3G and its variants hold great promise to be used in stem cell-based anti-HIV gene therapy clinical trials. Our work presented here takes a computational approach to give insights into the logistics of a successful A3G-SCT. Using the viral dynamics of each infected individual, we could also personalize the therapy to be highly effective. A3G-SCT may be an option for HIV patients undergoing bone marrow transplantation due to other complications such as AIDS-related lymphoma.

## Supporting Information

Method S1
**Estimation of the A3G-free Virus Release Ratio, 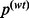, for WT CD4+ T Cells.**
(DOCX)

Method S2 **Model I: The Basic HIV Model for A3G-Augmented Cells (Reduced Burst Size for Cells Infected by A3G(+) Viruses).**
(DOCX)

Method S3 **Model Ib: The Basic HIV Model for A3G-Augmented Cells (Reduced Infectivity Rate for A3G(+) viruses).**
(DOCX)

Method S4 **Model IIa: The Basic HIV Model for WT and A3G-Augmented Cells.**
(DOCX)

Method S5 **Model IIb: The Basic HIV Model for WT and A3G-Augmented Cells with Lower Death Rates for Infected A3G-Augmented Cells.**
(DOCX)

Method S6 **Model IIc: The Basic HIV Model for WT and A3G-Augmented Cells with Lower Death Rates for Cells Infected by A3G(+) Viruses.**
(DOCX)

Method S7 **Model III: The Basic HIV Model for WT and A3G-Augmented Cells with Auto-Apoptosis Capability.**
(DOCX)

Method S8 **Model IV: The Basic HIV Model for A3G-Augmented Cells Overexpressing A3G at Low and High Levels.**
(DOCX)
