# Supplementary material for: APOBEC3G-Augmented Stem Cell Therapy to Modulate HIV Replication: A Computational Study
Source: PLoS One. 2013 May 22;8(5):e63984. doi: 10.1371/journal.pone.0063984 (PMC3661658; doi:10.1371/journal.pone.0063984)
Supplement: Method S1 — Estimation of the A3G-free Virus Release Ratio, , for WT CD4+ T Cells. (DOCX) [file pone.0063984.s001.docx]

# Estimation of the A3G-free Virus Release Ratio, *p*^(^*^wt^*^)^, for WT CD4+ T Cells

Refsland *et al*. recently generated a new cell line called CEM2n [[1](#_ENREF_1)]. The cell line is a diploid derivative of the tetraploid non-permissive human T cell line CEM. CEM2n expresses high levels of CD4 and CXCR4 and has non-permissive characteristics of CEM line. Using two rounds of rAAV-mediated gene targeting, they generated two A3G-null derivatives called A3GΔ1 and A3GΔ2 from CEM2n cell line. A3G-null derivatives and the parental cell line had similar growth rates. To evaluate the effect of A3G-knockout on HIV replication, cells were inoculated at a multiplicity of infection (MOI) of 1% with Vif-proficient HIV. Accumulation of p24 in the culture supernatants was monitored over time (Fig. S1 and [[1](#_ENREF_1)]).

We have previously developed a multicellular model describing an extracellular pool of HIV viruses infecting a population of T cells in cell culture [[2](#_ENREF_2)]. Using our model and experimental data from Refsland *et al*. [[1](#_ENREF_1)], we estimate the A3G-free virus release ratio for CEM2n cell line. In our simulations, the total transducing units (TU) can be calculated by multiplying MOI and total number of cells. It was estimated that 1 ng of p24 corresponds to 1000 transducing units [[3](#_ENREF_3)]. Assuming *T*_0_ = 500,000, we calculated the initial number of viruses at 5 ng p24. Since A3G-null cell lines do not express A3G, when they get infected they do not release A3G(+) viruses and hence *p*^(A3GΔ1)^ = *p*^(A3GΔ2)^ = 1. Using the data points corresponding to A3G-null cells (blue squares in Fig. S1), we found the optimum pair of burst size and virus infectivity rate such that the simulated HIV growth curves (blue lines) fit the experimental data with the minimum fitness error (Table S1). While keeping the estimated parameters fixed, we then used data points corresponding to CEM2n cells (red squares in Fig. S1) to estimate the A3G-free virus release ratio for these cells. Since Q-PCR results showed that CEM2n has roughly the same A3G mRNA expression as primary CD4+ T cells [[1](#_ENREF_1)], we use the average value of our estimates for *p*^(CEM2n)^ as the A3G-free virus release ratio for WT CD4+ T cells, i.e., *p*^(^*^wt^*^)^ = 0.83.

**Table S1.** Estimations of burst size, virus infectivity rate and A3G-free virus release ratio.

| **A3G-null cell line** | **Burst size** | **Virus infectivity rate** | ***p*^(CEM2n)^** |
| --- | --- | --- | --- |
| A3GΔ1 | 4100 | 26×10^-10^ | 0.81 |
| A3GΔ2 | 4100 | 28×10^-10^ | 0.85 |


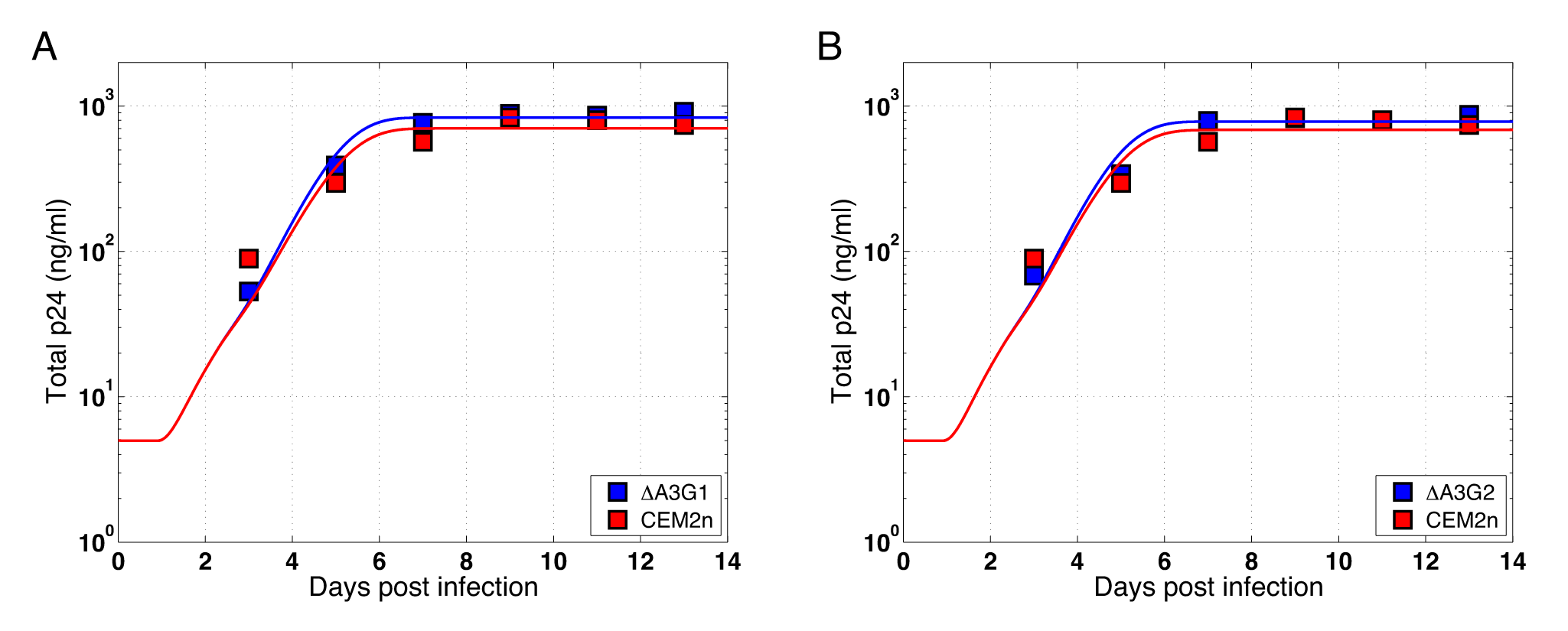


**Figure S1.** Blue squares represent p24 measurements in (A) A3GΔ1 and (B) A3GΔ1 cell lines after inoculation with Vif-proficient HIV at MOI = 0.01. Values of burst size and virus infectivity rate were estimated for each A3G-nul cell line by curve-fitting the blue data points to reach the minimum residual error. Keeping our estimates fixed for each cell line, we then estimated the value of *p*^(CEM2n)^ such that the generated red curve fitted the red data points with the minimum fitness error.

# References

1. Refsland EW, Hultquist JF, Harris RS (2012) Endogenous Origins of HIV-1 G-to-A Hypermutation and Restriction in the Nonpermissive T Cell Line CEM2n. PLoS Pathog 8: e1002800.

2. Hosseini I, Mac Gabhann F (2012) Multi-Scale Modeling of HIV Infection *in vitro* and APOBEC3G-Based Anti-Retroviral Therapy. PLoS Comput Biol 8: e1002371.

3. Zufferey R, Dull T, Mandel RJ, Bukovsky A, Quiroz D, et al. (1998) Self-Inactivating Lentivirus Vector for Safe and Efficient In Vivo Gene Delivery. Journal of Virology 72: 9873-9880.
