# Supplementary material for: APOBEC3G-Augmented Stem Cell Therapy to Modulate HIV Replication: A Computational Study
Source: PLoS One. 2013 May 22;8(5):e63984. doi: 10.1371/journal.pone.0063984 (PMC3661658; doi:10.1371/journal.pone.0063984)
Supplement: Method S2 — Model I: The Basic HIV Model for A3G-Augmented Cells (Reduced Burst Size for Cells Infected by A3G(+) Viruses). (DOCX) [file pone.0063984.s002.docx]

# Model I: The Basic HIV Model for A3G-Augmented Cells (Reduced Burst Size for Cells Infected by A3G(+) Viruses)

| 🡪 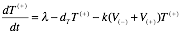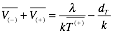 | (SI-1) |
| --- | --- |
| 🡪 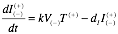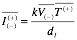 | (SI-2) |
| 🡪 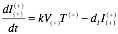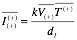 | (SI-3) |
| 🡪 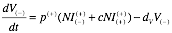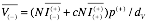 | (SI-4) |
| 🡪 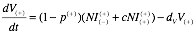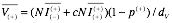 | (SI-5) |
| (SI-2) & (SI-4) 🡪 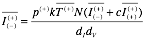 | (SI-6) |
| (SI-3) & (SI-5) 🡪 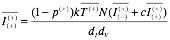 | (SI-7) |
| (SI-6) & (SI-7) 🡪 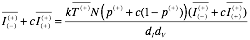 🡪 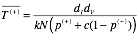 | (SI-8) |
| (SI-1) & (SI-8) 🡪🡪 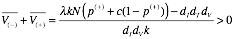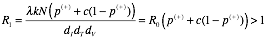 | (SI-9) |
