# Supplementary material for: APOBEC3G-Augmented Stem Cell Therapy to Modulate HIV Replication: A Computational Study
Source: PLoS One. 2013 May 22;8(5):e63984. doi: 10.1371/journal.pone.0063984 (PMC3661658; doi:10.1371/journal.pone.0063984)
Supplement: Method S3 — Model Ib: The Basic HIV Model for A3G-Augmented Cells (Reduced Infectivity Rate for A3G(+) viruses). (DOCX) [file pone.0063984.s003.docx]

# Model Ib: The Basic HIV Model for A3G-Augmented Cells (Reduced Infectivity Rate for A3G(+) viruses)

| 🡪 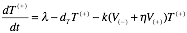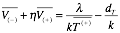 | (SIb-1) |
| --- | --- |
| 🡪 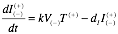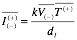 | (SIb-2) |
| 🡪 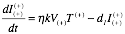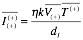 | (SIb-3) |
| 🡪 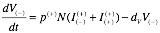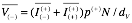 | (SIb-4) |
| 🡪 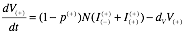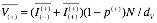 | (SIb-5) |
| (SIb-2) & (SIb-4) 🡪 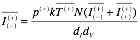 | (SIb-6) |
| (SIb-3) & (SIb-5) 🡪 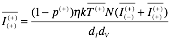 | (SIb-7) |
| (SIb-6) & (SIb-7) 🡪 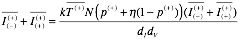 🡪 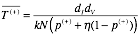 | (SIb-8) |
| (SIb-1) & (SIb-8) 🡪🡪 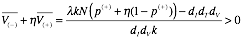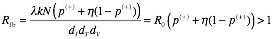 | (SIb-9) |
