# Supplementary material for: APOBEC3G-Augmented Stem Cell Therapy to Modulate HIV Replication: A Computational Study
Source: PLoS One. 2013 May 22;8(5):e63984. doi: 10.1371/journal.pone.0063984 (PMC3661658; doi:10.1371/journal.pone.0063984)
Supplement: Method S4 — Model IIa: The Basic HIV Model for WT and A3G-Augmented Cells. (DOCX) [file pone.0063984.s004.docx]

# Model IIa: The Basic HIV Model for WT and A3G-Augmented Cells

| 🡪 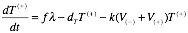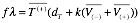 | (SIIa-1) |
| --- | --- |
| 🡪 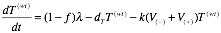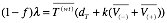 | (SIIa-2) |
| 🡪 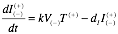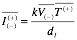 | (SIIa-3) |
| 🡪 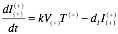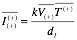 | (SIIa-4) |
| 🡪 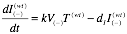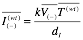 | (SIIa-5) |
| 🡪 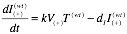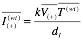 | (SIIa-6) |
| 🡪 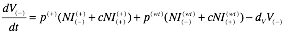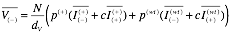 | (SIIa-7) |
| 🡪 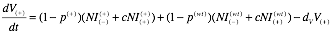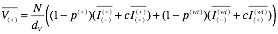 | (SIIa-8) |
| (SIIa-3) & (SIIa-7) 🡪 where 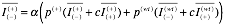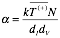 | (SIIa-9) |
| (SIIa-4) & (SIIa-8) 🡪 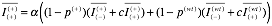 | (SIIa-10) |
| (SIIa-5) & (SIIa-7) 🡪 where 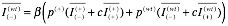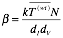 | (SIIa-11) |
| (SIIa-6) & (SIIa-8) 🡪 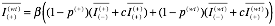 | (SIIa-12) |
| (SIIa-1) & (SIIa-2) 🡪 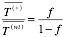 | (SIIa-13) |
| (SIIa-9) & (SIIa-11) 🡪 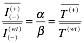 | (SIIa-14) |
| (SIIa-10) & (SIIa-12) 🡪 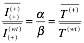 | (SIIa-15) |
| (SIIa-9) & (SIIa-10) & (SIIa-14) & (SIIa-15) 🡪 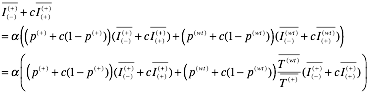 🡪 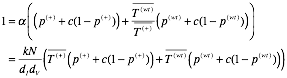 | (SIIa-16) |
| (SIIa-13) & (SIIa-16) 🡪 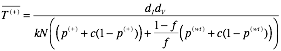 | (SIIa-17) |
| (SIIa-1) & (SIIa-17) 🡪 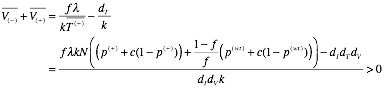 🡪 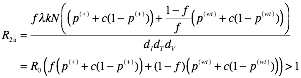 | (SIIa-18) |
