# Supplementary material for: APOBEC3G-Augmented Stem Cell Therapy to Modulate HIV Replication: A Computational Study
Source: PLoS One. 2013 May 22;8(5):e63984. doi: 10.1371/journal.pone.0063984 (PMC3661658; doi:10.1371/journal.pone.0063984)
Supplement: Method S5 — Model IIb: The Basic HIV Model for WT and A3G-Augmented Cells with Lower Death Rates for Infected A3G-Augmented Cells. (DOCX) [file pone.0063984.s005.docx]

# Model IIb: The Basic HIV Model for WT and A3G-Augmented Cells with Lower Death Rates for Infected A3G-Augmented Cells

| 🡪 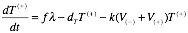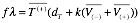 | (SIIb-1) |
| --- | --- |
| 🡪 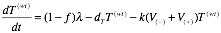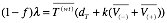 | (SIIb-2) |
| 🡪 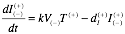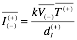 | (SIIb-3) |
| 🡪 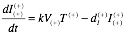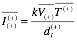 | (SIIb-4) |
| 🡪 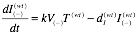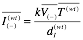 | (SIIb-5) |
| 🡪 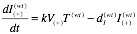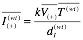 | (SIIb-6) |
| 🡪 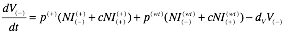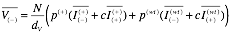 | (SIIb-7) |
| 🡪 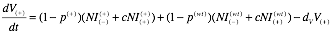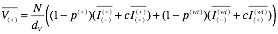 | (SIIb-8) |
| (SIIb-3) & (SIIb-7) 🡪 where 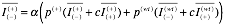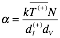 | (SIIb-9) |
| (SIIb-4) & (SIIb-8) 🡪 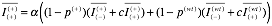 | (SIIb-10) |
| (SIIb-5) & (SIIb-7) 🡪 where 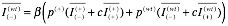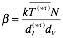 | (SIIb-11) |
| (SIIb-6) & (SIIb-8) 🡪 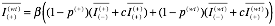 | (SIIb-12) |
| (SIIb-1) & (SIIb-2) 🡪 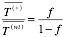 | (SIIb-13) |
| (SIIb-9) & (SIIb-11) 🡪 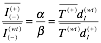 | (SIIb-14) |
| (SIIb-10) & (SIIb-12) 🡪 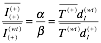 | (SIIb-15) |
| (SIIb-9) & (SIIb-10) & (SIIb-14) & (SIIb-15) 🡪 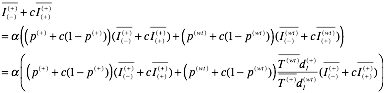 🡪 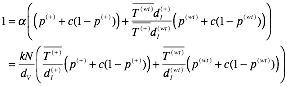 | (SIIb-16) |
| (SIIb-13) & (SIIb-16) 🡪 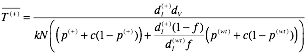 | (SIIb-17) |
| (SIIb-1) & (SIIb-17) 🡪 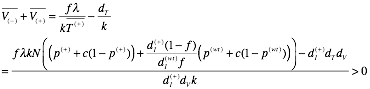 🡪 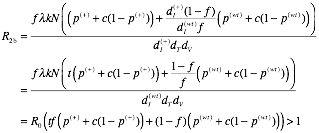 where 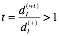 | (SIIb-18) |
