# Supplementary material for: APOBEC3G-Augmented Stem Cell Therapy to Modulate HIV Replication: A Computational Study
Source: PLoS One. 2013 May 22;8(5):e63984. doi: 10.1371/journal.pone.0063984 (PMC3661658; doi:10.1371/journal.pone.0063984)
Supplement: Method S6 — Model IIc: The Basic HIV Model for WT and A3G-Augmented Cells with Lower Death Rates for Cells Infected by A3G(+) Viruses. (DOCX) [file pone.0063984.s006.docx]

# Model IIc: The Basic HIV Model for WT and A3G-Augmented Cells with Lower Death Rates for Cells Infected by A3G(+) Viruses

| 🡪 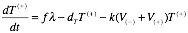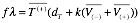 | (SIIc-1) |
| --- | --- |
| 🡪 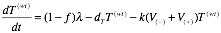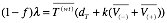 | (SIIc-2) |
| 🡪 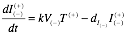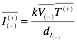 | (SIIc-3) |
| 🡪 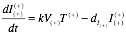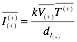 | (SIIc-4) |
| 🡪 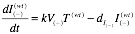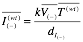 | (SIIc-5) |
| 🡪 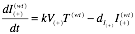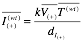 | (SIIc-6) |
| 🡪 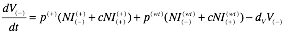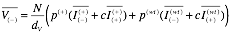 | (SIIc-7) |
| 🡪 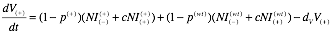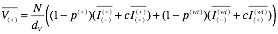 | (SIIc-8) |
| (SIIc-3) & (SIIc-7) 🡪 where 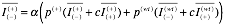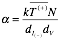 | (SIIc-9) |
| (SIIc-4) & (SIIc-8) 🡪 where 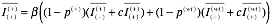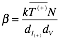 | (SIIc-10) |
| (SIIc-5) & (SIIc-7) 🡪 where 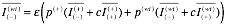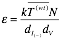 | (SIIc-11) |
| (SIIc-6) & (SIIc-8) 🡪 where 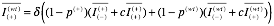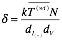 | (SIIc-12) |
| (SIIc-1) & (SIIc-2) 🡪 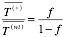 | (SIIc-13) |
| (SIIc-9) & (SIIc-11) 🡪 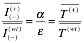 | (SIIc-14) |
| (SIIc-10) & (SIIc-12) 🡪 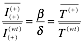 | (SIIc-15) |
| (SIIc-9) & (SIIc-10) & (SIIc-14) & (SIIc-15) 🡪 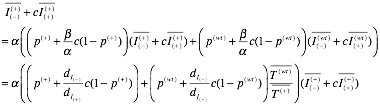 🡪 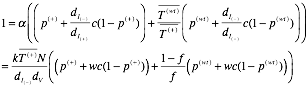 | (SIIc-16) |
| (SIIc-13) & (SIIc-16) 🡪 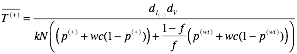 | (SIIc-17) |
| (SIIc-1) & (SIIc-17) 🡪 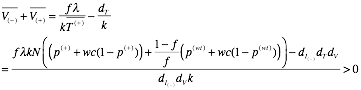 🡪 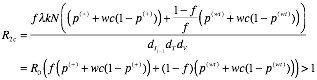 where 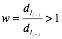 | (SIIc-18) |
