# Supplementary material for: APOBEC3G-Augmented Stem Cell Therapy to Modulate HIV Replication: A Computational Study
Source: PLoS One. 2013 May 22;8(5):e63984. doi: 10.1371/journal.pone.0063984 (PMC3661658; doi:10.1371/journal.pone.0063984)
Supplement: Method S7 — Model III: The Basic HIV Model for WT and A3G-Augmented Cells with Auto-Apoptosis Capability. (DOCX) [file pone.0063984.s007.docx]

# Model III: The Basic HIV Model for WT and A3G-Augmented Cells with Auto-Apoptosis Capability

| 🡪 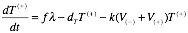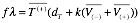 | (SIII-1) |
| --- | --- |
| 🡪 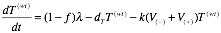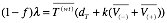 | (SIII-2) |
| 🡪 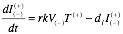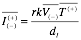 | (SIII-3) |
| 🡪 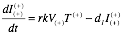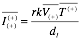 | (SIII-4) |
| 🡪 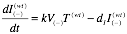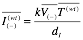 | (SIII-5) |
| 🡪 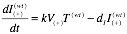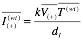 | (SIII-6) |
| 🡪 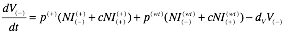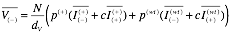 | (SIII-7) |
| 🡪 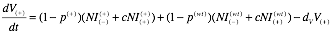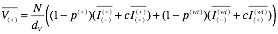 | (SIII-8) |
| (SIII-3) & (SIII-7) 🡪 where 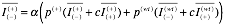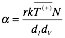 | (SIII-9) |
| (SIII-4) & (SIII-8) 🡪 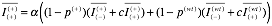 | (SIII-10) |
| (SIII-5) & (SIII-7) 🡪 where 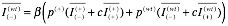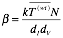 | (SIII-11) |
| (SIII-6) & (SIII-8) 🡪 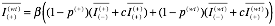 | (SIII-12) |
| (SIII-1) & (SIII-2) 🡪 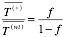 | (SIII-13) |
| (SIII-9) & (SIII-11) 🡪 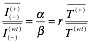 | (SIII-14) |
| (SIII-10) & (SIII-12) 🡪 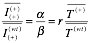 | (SIII-15) |
| (SIII-9) & (SIII-10) & (SIII-14) & (SIII-15) 🡪 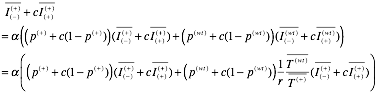 🡪 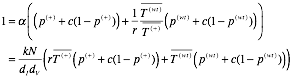 | (SIII-16) |
| (SIII-13) & (SIII-16) 🡪 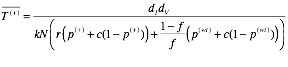 | (SIII-17) |
| (SIII-1) & (SIII-17) 🡪 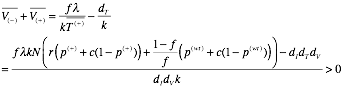 🡪 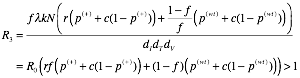 | (SIII-18) |
