# Supplementary material for: APOBEC3G-Augmented Stem Cell Therapy to Modulate HIV Replication: A Computational Study
Source: PLoS One. 2013 May 22;8(5):e63984. doi: 10.1371/journal.pone.0063984 (PMC3661658; doi:10.1371/journal.pone.0063984)
Supplement: Method S8 — Model IV: The Basic HIV Model for A3G-Augmented Cells Overexpressing A3G at Low and High Levels. (DOCX) [file pone.0063984.s008.docx]

# Model IV: The Basic HIV Model for A3G-Augmented Cells Overexpressing A3G at Low and High Levels

| 🡪 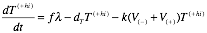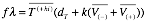 | (SIV-1) |
| --- | --- |
| 🡪 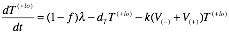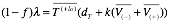 | (SIV-2) |
| 🡪 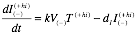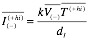 | (SIV-3) |
| 🡪 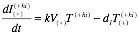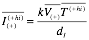 | (SIV-4) |
| 🡪 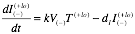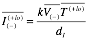 | (SIV-5) |
| 🡪 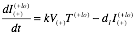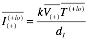 | (SIV-6) |
| 🡪 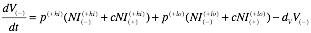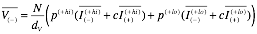 | (SIV-7) |
| 🡪 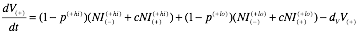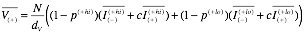 | (SIV-8) |
| (SIV-3) & (SIV-7) 🡪 where 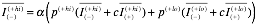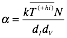 | (SIV-9) |
| (SIV-4) & (SIV-8) 🡪 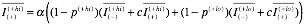 | (SIV-10) |
| (SIV-5) & (SIV-7) 🡪 where 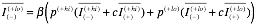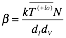 | (SIV-11) |
| (SIV-6) & (SIV-8) 🡪 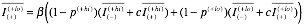 | (SIV-12) |
| (SIV-1) & (SIV-2) 🡪 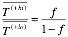 | (SIV-13) |
| (SIV-9) & (SIV-11) 🡪 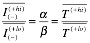 | (SIV-14) |
| (SIV-10) & (SIV-12) 🡪 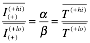 | (SIV-15) |
| (SIV-9) & (SIV-10) & (SIV-14) & (SIV-15) 🡪 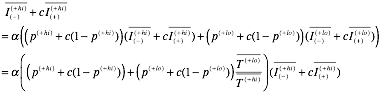 🡪 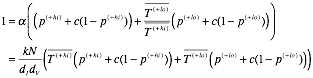 | (SIV-16) |
| (SIV-13) & (SIV-16) 🡪 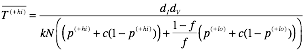 | (SIV-17) |
| (SIV-1) & (SIV-17) 🡪 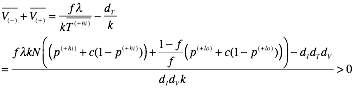 🡪 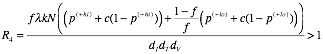 | (SIV-18) |
